# JAXA’s new high-resolution land use land cover map for Vietnam using a time-feature convolutional neural network

**DOI:** 10.1038/s41598-024-54308-1

**Published:** 2024-02-16

**Authors:** Van Thinh Truong, Sota Hirayama, Duong Cao Phan, Thanh Tung Hoang, Takeo Tadono, Kenlo Nishida Nasahara

**Affiliations:** 1 Degree Programs in Life and Earth Sciences, Graduate School of Science and Technology, University of Tsukuba, Tennoudai 1-1-1, Tsukuba, Ibaraki 305-8572 Japan; 2 Earth Observation Research Center (EORC), Japan Aerospace Exploration Agency (JAXA), Sengen 2-1-1, Tsukuba, Ibaraki 305-8505 Japan; 3 Ireland’s Centre For Applied AI, School of Computer Science, University College Dublin, Dublin 4, D02 V2N9, Belfield, Ireland; 4 Hydraulic Construction Institute, Vietnam Academy for Water Resources, No. 3, Alley 95, Chua Boc Street, Dong Da District, Hanoi 116765 Vietnam; 5 Faculty of International Studies, Hanoi University, Km 9, Nguyen Trai Road, Nam Tu Liem District, Hanoi 100000 Vietnam; 6 Faculty of Life and Environmental Sciences, University of Tsukuba, Tennoudai 1-1-1, Tsukuba, Ibaraki 305-8572 Japan

**Keywords:** Environmental impact, Sustainability

## Abstract

Land use land cover (LULC) maps are crucial for various applications, such as disaster management, natural resource conservation, biodiversity evaluation, climate modeling, etc. The Japan Aerospace Exploration Agency (JAXA) has released several high-resolution LULC maps for national and regional scales. Vietnam, due to its rich biodiversity and cultural diversity, is a target country for the production of high-resolution LULC maps. This study introduces a high-resolution and high-accuracy LULC map for Vietnam, utilizing a CNN approach that performs convolution over a time-feature domain instead of the typical geospatial domain employed by conventional CNNs. By using multi-temporal data spanning 6 seasons, the produced LULC map achieved a high overall accuracy of 90.5% ± 1.2%, surpassing other 10-meter LULC maps for Vietnam in terms of accuracy and/or the ability to capture detailed features. In addition, a straightforward and practical approach was proposed for generating cloud-free multi-temporal Sentinel-2 images, particularly suitable for cloudy regions. This study marks the first implementation of the time-feature CNN approach for the creation of a high-accuracy LULC map in a tropical cloudy country.

## Introduction

Land use land cover (LULC) maps provide valuable information for understanding different anthropogenic-related processes, including climate change^1^, urban expansion^2^, urban heat island^3^, and sediment-related disasters^4^. Recently, several high-resolution global LULC products have been released, such as Google’s Dynamic World (DW)^5^, ESA’s World Cover (ESA)^6^, and ESRI Land Cover (ESRI)^7^. These LULC products have garnered the widespread attention due to their high spatial resolution of 10 m and their capability to provide a time series of LULC maps on a global scale. While these recent global products have significantly improved the spatial detail of LULC maps, their applicability at national scales is limited due to coarse LULC category systems and uncertainties in accuracy when applied to national or local scales. For example, in the aforementioned global LULC maps, all forest types were combined into a single category such as “tree”. Users requiring more detailed forest categorization cannot use these global maps for their work. Given these limitations, the creation of national LULC maps becomes necessary. Several studies have undertaken the mapping of LULC in response to national or regional demands, including the production of high-resolution LULC maps of Japan^8,9^, LULC maps of China for the years 1980-2015^10^, a 10-m European LULC map^11^, and a LULC map of West Africa^12^.

In recent years, deep learning has gained significant traction in remote-sensing-related studies^13^, driven by technological advancements, such as data augmentation, non-linear activation, high-performance graphics processing unit (GPU), and cloud computing^14^. Several studies have indicated that deep learning consistently outperformed shallow machine learning methods in terms of overall accuracy^15,16^. For instance, deep learning methods achieved impressive results, with a median accuracy of approximately 95% for scene classification, around 92% for object detection, and about 91% for LULC classification using benchmark datasets^17^. Among the deep learning methods, Convolutional Neural Networks (CNNs) have emerged as one of the most popular and powerful algorithms for satellite remote sensing applications, particularly in LULC classification^18^. The compatibility of CNNs with satellite images can be attributed to their design, which is well-suited for processing multi-dimensional arrays^19^. Consequently, numerous studies have successfully applied CNNs to map LULC using various types of satellite images, including high-resolution^20–22^, medium-resolution^23–25^, and hyperspectral images^26–28^. A review of 200 papers on remote sensing applications using deep learning by Ma et al. (2019)^17^ revealed that most of LULC classification studies focused on high-resolution ($\le$ 10 m) and hyperspectral images to harness their rich spatial or spectral information. Surprisingly, medium-resolution images (e.g., 30 m) received less attention despite their popularity and accessibility. Additionally, the review highlighted that the majority of studies relied on single images, with only a few attempting to analyze multi-temporal remote sensing data for LULC classification using CNNs. The limitation of applying CNNs to multi-temporal data is primarily due to their inherent design, which mainly processes single images. In response to this limitation, some studies have explored alternative approaches, such as Recurrent Neural Networks (RNNs)^29,30^, or hybrid models combining CNNs and RNNs for LULC classification^31–33^. However, these approaches often entail high computational costs and complex model structures, which can pose challenges for large-scale LULC mapping efforts.

On the other hand, the spatial convolution process, when combined with down-sampling methods like max-pooling, presents challenges due to information loss and reduced spatial resolution. To tackle these challenges, numerous studies have explored different approaches. Some have sought to simplify the standard Convolutional Neural Network (CNN) architecture by employing a homogeneous network consisting exclusively of convolutional layers, coupled with dimensionality reduction through increased stride^34^. Others have focused on multi-scale context aggregation, achieved through dilated convolutions without pooling or sub-sampling^35^. Additionally, there have been attempts to employ deconvolutional networks to recover spatial resolution compromised by down-sampling^36^. These studies have introduced valuable approaches to address information loss-related challenges, primarily in the context of scene classification or object detection tasks. However, the application of these methods to pixel-level classification, particularly in land use land cover (LULC) classification using satellite images, remains uncertain.

To overcome the limitations of conventional Convolutional Neural Networks (CNNs) in maintaining spatial details of LULC maps, Hirayama et al. (2022) introduced a software package, namely SACLASS2^9^ that employs multitemporal satellite images with a standard CNN architecture for LULC mapping. SACLASS2 was used to create a 12-category LULC map for Japan with an overall accuracy of $88.85\%$, surpassing recent LULC products for Japan in terms of spatial detail. In Hirayama’s method, a time series of images was generated for four seasons, encompassing winter, spring, summer, and autumn. The seasonal data were cleverly organized in a manner that enabled the extraction of both temporal and feature (bands and indices) information from individual pixels. Notably, this approach offers flexibility in the selection of satellite images and exhibits substantial potential for large-scale LULC mapping. However, a comprehensive exploration of its capabilities is insufficient, signifying a need for further research to fully tap into the potential of this approach.

Vietnam has attracted considerable attention from numerous international organizations for biodiversity and environmental conservation, primarily due to its remarkable levels of biodiversity, cultural diversity, and rapid economic development. In fact, Vietnam was ranked in the top 16 countries globally in terms of biodiversity richness^37^. The country is home to 54 ethnic groups, each contributing to its rich cultural heritage and distinct land use practices. However, the country faces pressing challenges due to a critical rate of biodiversity loss^38^ and environmental degradation^39^. These environmental issues are primarily attributed to the consequences of rapid population growth and economic expansion witnessed in recent decades. Among the direct contributors, the change in LULC stands out as one of the most significant issues. LULC change has led to the degradation and loss of critical ecosystems and habitats, endangering numerous species, causing degradation of natural resources, and triggering natural disasters. Hence, it becomes imperative to regularly map LULC on a national scale. This mapping effort plays a pivotal role in providing crucial information for biodiversity evaluation and natural disaster countermeasures for Vietnam.

JAXA has released several LULC products for Mainland Vietnam, including 50-m LULC maps from 2015 to 2018^40^, 10-m LULC maps for 2007 and 2016^41^, 30-m LULC maps from 1990 to 2020^42^. This study is part of a series of research efforts focused on enhancing JAXA’s LULC mapping for Vietnam. Our objective is to create a high-resolution and high-accuracy LULC map by adopting and extending Hirayama’s SACLASS2 software to incorporate 6 seasons of satellite data. In this paper, Hirayama’s approach is denoted as the “time-feature CNN” approach. The contributions of this paper can be summarized as follows: (1) Providing a new and reliable LULC map for Vietnam by employing the time-feature CNN approach with the integration of 6 seasons of satellite data; (2) Proposing a straightforward and practical method for constructing a composite dataset of 6 seasons for Sentinel-2, facilitating its application in tropical, cloudy countries.

The remainder of this paper is organized in the following order: study area, results, discussions, and methods and materials.

## Study area

**Figure 1:**
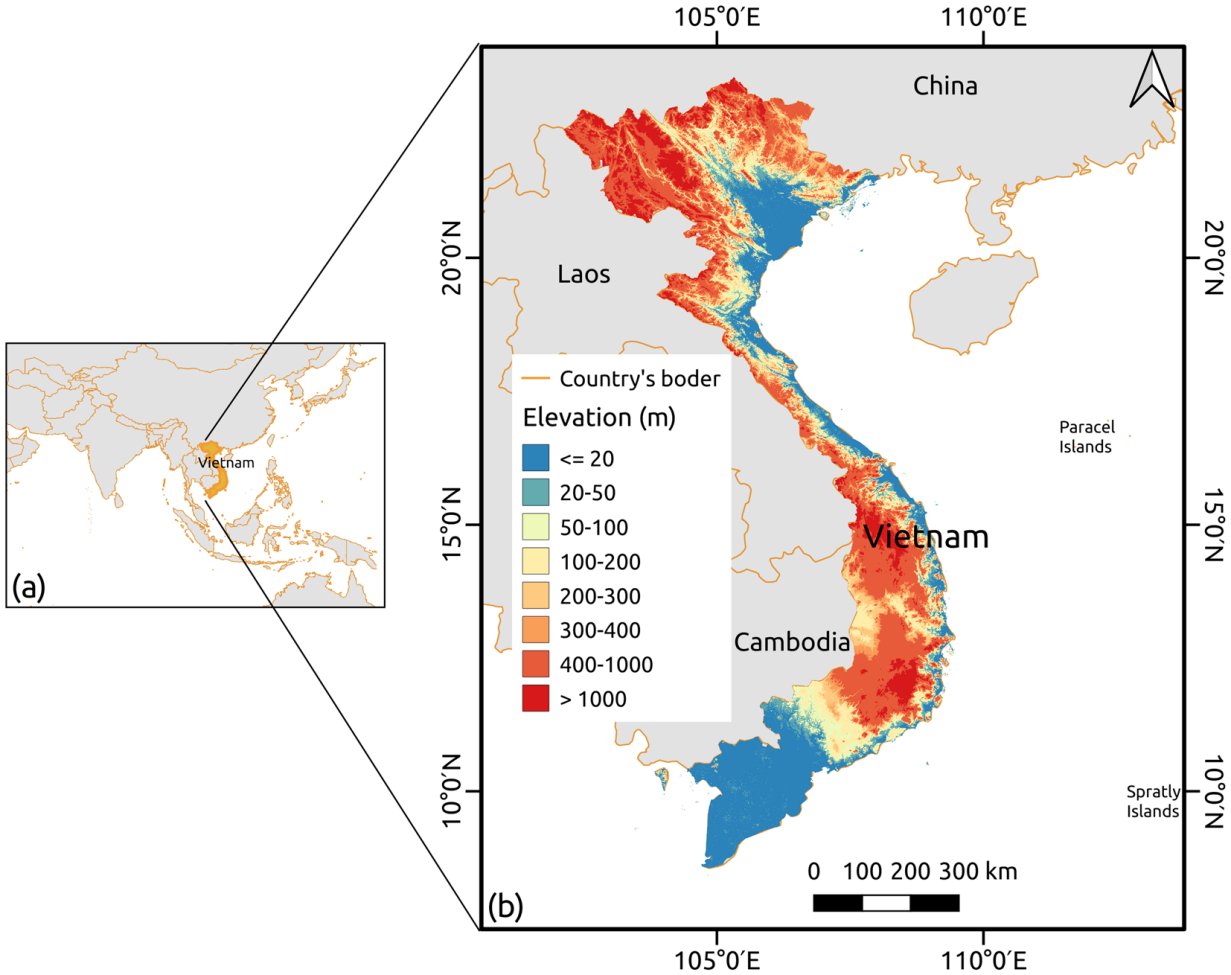
The study area is Mainland Vietnam, excluding some remote islands in the east. (**a**) The location of Vietnam on the world map, (**b**) The elevation map of Mainland Vietnam. A large area is covered by high mountains (> 1000 m). The flat lands are distributed along the coastal line (blue areas) and the two big deltas provide a majority of agricultural products for the country.

The study area encompasses Mainland Vietnam, covering approximately about 324,000 km^2^. Mainland Vietnam is characterized by diverse landscapes, featuring high mountains exceeding 1000 m in elevation, as well as hilly terrain that stretches from the northwest to the southeast. These highlands occupy about three-quarters of the total area. The remaining one-quarter of the land consists of low-lying areas, including the Red River Delta in the north and the Mekong River Delta in the south. These lowlands are complemented by flat coastal regions (see Fig. 1). Vietnam experiences a varied climate, transitioning from subtropical in the north to tropical in the south. The rainy or wet season, often referred to as the cloudy season, prevails from May to October. Since the economic revolution in 1986, Vietnam has undergone rapid changes in land use and land cover. Recent years have witnessed a swift urbanization process, resulting in the transformation of numerous farmlands and green areas into urban lands^43^. Despite this urbanization trend, Vietnam has managed to achieve a transition from net forest loss to net gain, primarily driven by the expansion of plantation forests through reforestation programs in recent decades^44,45^.

## Results

### Time series of Sentinel-2 images

To generate cloud-free Sentinel-2 composite images for the 6 seasons in 2020, we employed a straightforward approach that incorporated data from 2019, 2020, and 2021. The inclusion of data from both 2019 and 2021 served a dual purpose: it addressed data gaps caused by cloud masking and eliminated pixels with abnormal values. A comprehensive description of our approach can be found in the “Satellite images pre-processing” section. After a meticulous assessment of the resulting images, most of missing data were successfully replaced by valid data (refer to the link provided in the “Data Availability” section). While a few pixels with missing data persisted in the final images, addressing these would require a more intricate solution. At this stage, we accepted these minor data gaps for the sake of maintaining the simplicity and the practicality in our approach, particularly since Synthetic Aperture Radar (SAR) data would subsequently compensate for any missing optical data.

**Figure 2:**
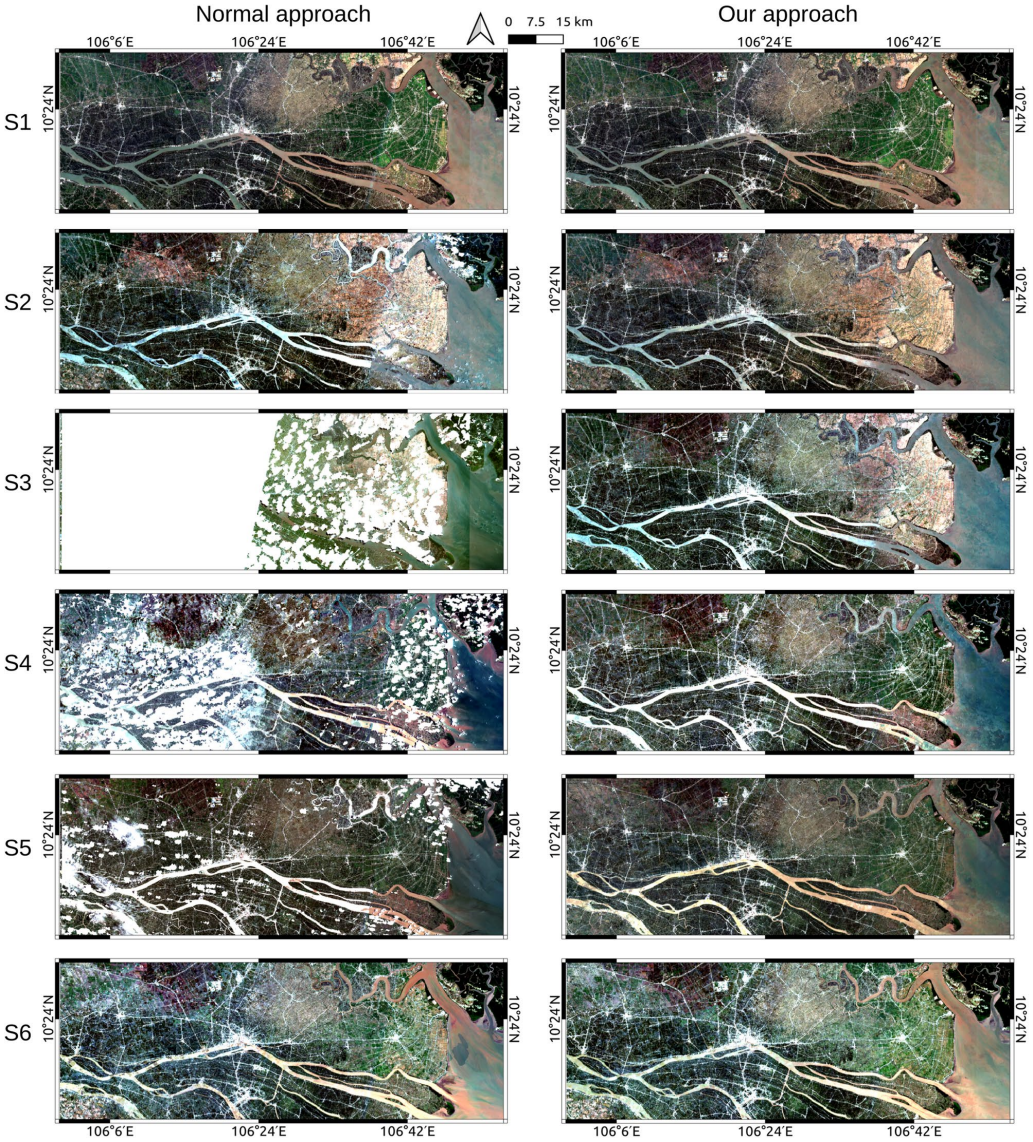
Comparison between the normal approach and our approach for creating a time series of Sentinel-2 images with 6 seasons (S1-S6), an example for a part of Mekong River Delta in Vietnam. All images of 6 seasons, both for the normal approach and our approach, were produced using free Sentinel-2 level 2A images provided by ESA via Google Earth Engine^46^, by applying the “median()” method available on Google Earth Engine^47^. This figure was organized and presented using QGIS 3.10.4-A Coruna (https://qgis.org/en/site/).

Figure 2 illustrates the enhancements achieved in Sentinel-2 images through our approach when compared to the conventional method (the normal approach). The improvements can be described in two main aspects: (1) Our approach effectively addressed the missing data problem by leveraging information from other years of the same season or from different seasons in the same year; (2) This approach successfully reduced the presence of undesirable pixels, such as cloudy pixels or abnormal values pixels due to incompleteness of the atmospheric correction. In the normal approach, clouds led to substantial missing data from Season 3 (S3) to Season 5 (S5). Conversely, our approach effectively filled these missing data.

### LULC map of Mainland Vietnam

**Figure 3:**
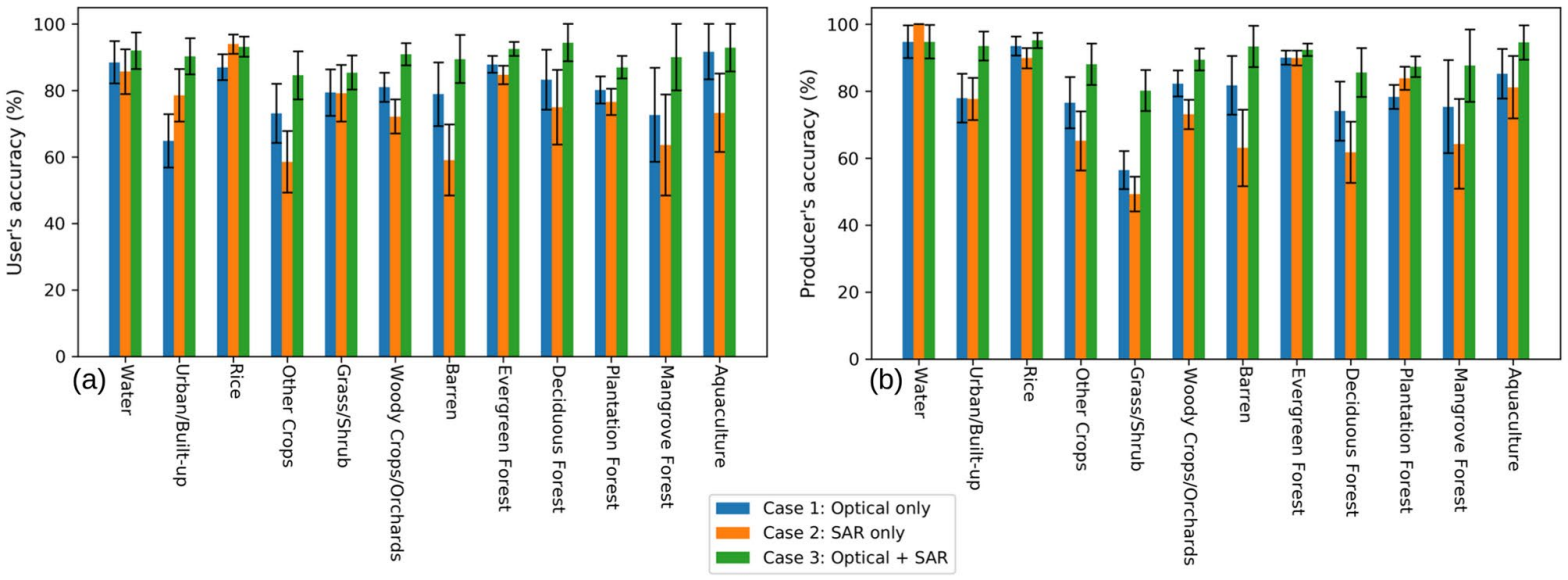
Comparison of accuracy for each category in three cases. (**a**) User’s accuracy. (**b**) Producer’s accuracy.

**Figure 4:**
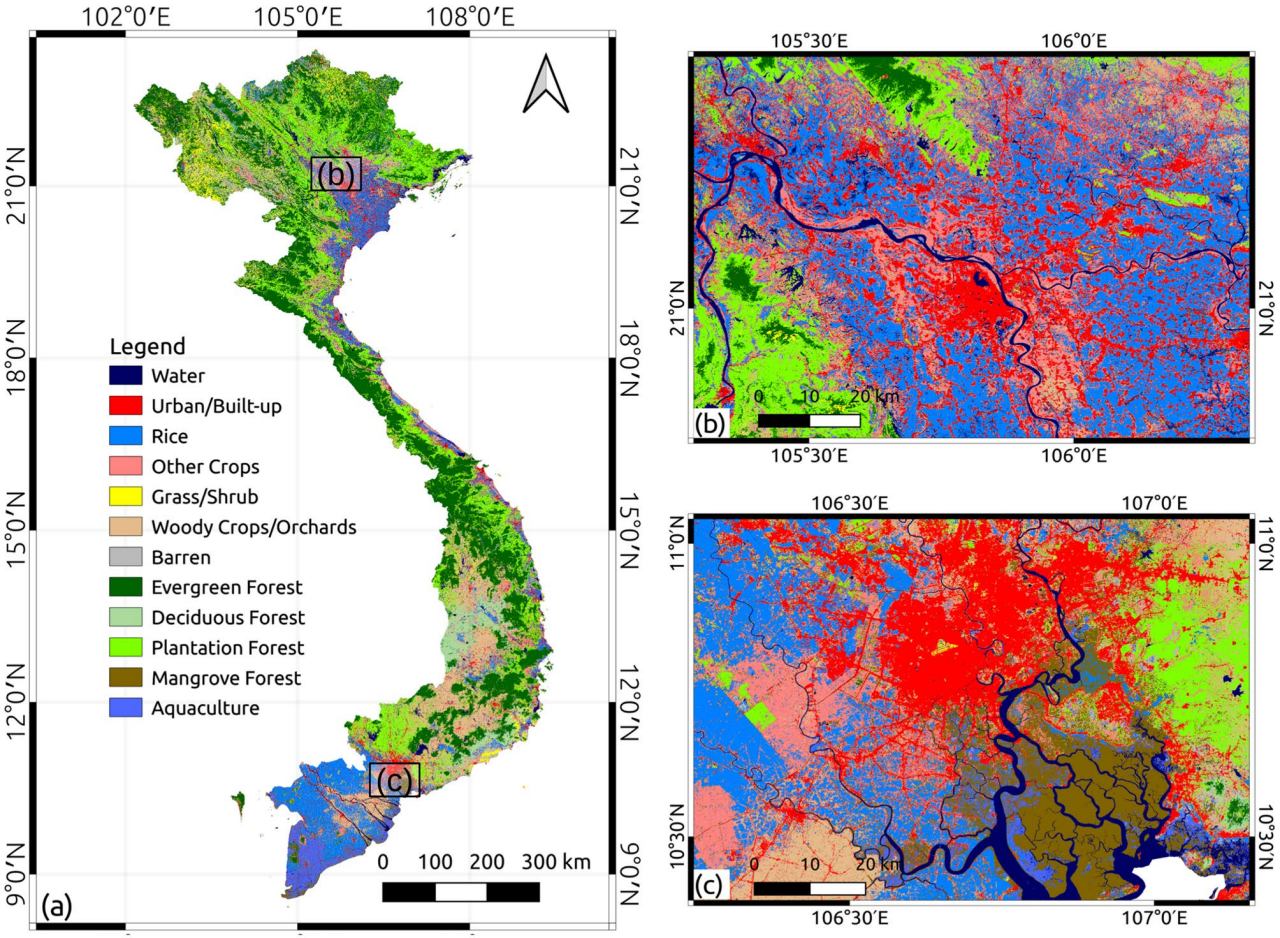
The classified LULC map for Vietnam. (**a**) The classified LULC map for Vietnam in Case 3. (**b**) A zoom-in of (**a**) for Hanoi City and surrounding areas. (**c**) A zoom-in of (**a**) for Ho Chi Minh City and the surrounding areas.

By fusing multi-temporal optical and SAR images, our LULC map (see Fig. 4) achieved an impressive overall accuracy of $90.5\% \pm 1.2\%$ (see Supplementary Table S6). The average user’s and producer’s accuracy are also notably high, standing at $90.2\% \pm 1.7\%$ and $90.2\% \pm 1.6\%$, respectively. Nearly all categories exhibited an accuracy rate exceeding 85% for both user’s and producer’s accuracy. Specifically, the categories of water, urban/built-up, and rice demonstrated the highest user’s and producer’s accuracy, while other crops and grass/shrub categories exhibited the lowest accuracy (see Fig. 3). The lower accuracy in the other crops and grass/shrub categories may be attributed to the intra-category variability. Agriculture in Vietnam is well-known for its heterogeneous and fragmented lands, characterized by small-scale farming. This fact caused a diverse range of crop types with various cultivation practices, creating a higher possibility of misclassification from crops into other categories.

Within the forest category, there were misclassifications between evergreen forests and plantation forests. Plantation forests are typically characterized by the homogeneity of plant types and a cycle of harvesting. However, the presence of vigorous understory vegetation, common in tropical climates, along with the extended harvesting cycle spanning 10 to 20 years, results in plantation forests that are notably dense and appear very dark in satellite images. Consequently, this density and darkness sometimes lead to the misclassification of plantation forests as natural forests in satellite imagery.

To examine the performance of the classification method with different combinations of input images, a comparison was implemented between the resulting LULC map generated by using only optical images (Case 1), using only SAR images (Case 2), and fusing optical and SAR images (Case 3). The specifics of image selection are provided in Table 1.

**Table 1:** The data utilized for LULC classification in three cases. The feature numbers and time series information are detailed in Fig. 9.

**Case**	**Feature**	**Time series**
Case 1 (Optical only)	1-17 & 36-40	S1-S6
Case 2 (SAR only)	18-40	S1-S6
Case 3 (Optical + SAR)	1-40	S1-S6

**Figure 5:**
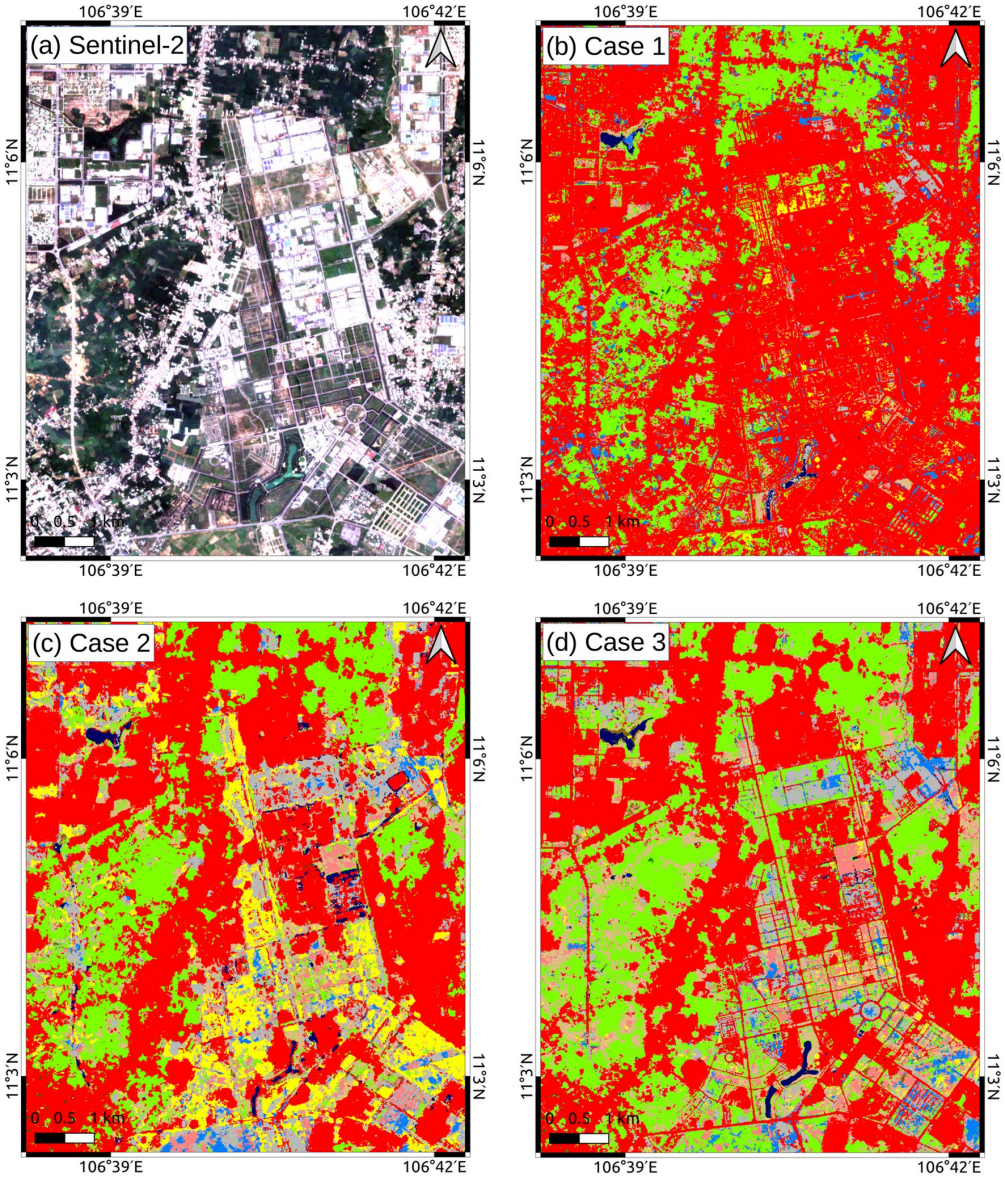
Comparison of LULC maps for three cases. (**a**) Sentinel-2 RGB image, (**b**) LULC map for Case 1 (optical only), (**c**) LULC map for Case 2 (SAR only), (**d**) LULC for Case 3 (optical + SAR). The true-color satellite image in (**a**) was produced using the Sentinel-2 level 2A image (Red: band 4, Green: band 3, and Blue: band 2), provided for free by ESA via Google Earth Engine^46^. The maps in (**b**), (**c**) and (**d**) are classified LULC maps using SACLASS-2 software version 1.0 produced by Hirayama et al. (2022)^9^. This figure was organized and presented using QGIS 3.10.4-A Coruna (https://qgis.org/en/site/).

The same CNN structure was employed (see Fig. 10) for all three cases, with the same number of training data (121,521 points). Our accuracy assessment for three cases relied on a validation dataset of 600 points described in the “Methods and materials” section. The results revealed a significant improvement in both the user’s and producer’s accuracy for most of the categories when combining optical and SAR data (see Fig. 3 and Supplementary Table S1–S6). Using only SAR images produced a LULC map with the lowest overall accuracy of $79.6\% \pm 1.6\%$, while employing only optical data produced an overall accuracy of $82.7\% \pm 1.5\%$. Remarkably, the integration of optical and SAR images achieved the highest overall accuracy of $90.5\% \pm 1.2\%$. Figure 5 visually compares the classification results for three cases to a true-color Sentinel-2 image. In Case 1, the misclassification of grass/shrub and barren to urban/built-up areas caused overestimation for this category (see Fig. 5b). Case 2 improved urban/built-up area classification compared to Case 1, but finer objects like narrow roads remained a challenge (see Fig. 5c). Notably, Case 3 outperformed the other cases, offering a clear representation of road networks. The fusion of optical and SAR data not only resolved misclassifications present in the optical-only case but also accurately identified small features (see Fig. 5d).

### Comparison with other LULC products

For the purpose of comparing with our LULC map, this study selected available 10-m LULC maps for Vietnam, including three global LULC products for 2020 (DW, ESA, and ESRI), and one national LULC product from 2016 released by JAXA. To obtain DW’s LULC map, we computed the mode of all daily LULC maps of DW in 2020 for the “label” band using Google Earth Engine. ESRI’s, ESA’s, and JAXA’s product can be directly downloaded from their respective websites. To facilitate a meaningful comparison among these different LULC products, all the maps were reclassified to a common LULC category system, consisting of 7 categories: (1) water, (2) urban/built-up, (3) tree, (4) barren, (5) grass/shrub, (6) flooded vegetation (including mangrove trees), and (7) crops. Following this reclassification, the accuracy assessment was performed for the global LULC maps using the validation dataset comprising 600 points, as described in the “Methods and materials” section. The overall accuracy and its standard deviation for JAXA’s 2016 map were calculated based on the original error matrix provided with the LULC map on the JAXA’s website^48^. Our map achieved an overall accuracy of 93.2% ± 1.7%, surpassing DW with 80.1% ± 2.5%, ESA with 84.3% ± 2.3%, ESRI with 71.6% ± 2.9%, and JAXA’s 2016 map with 88.4% ± 0.6% (see Supplementary Table S7-S16).

**Figure 6:**
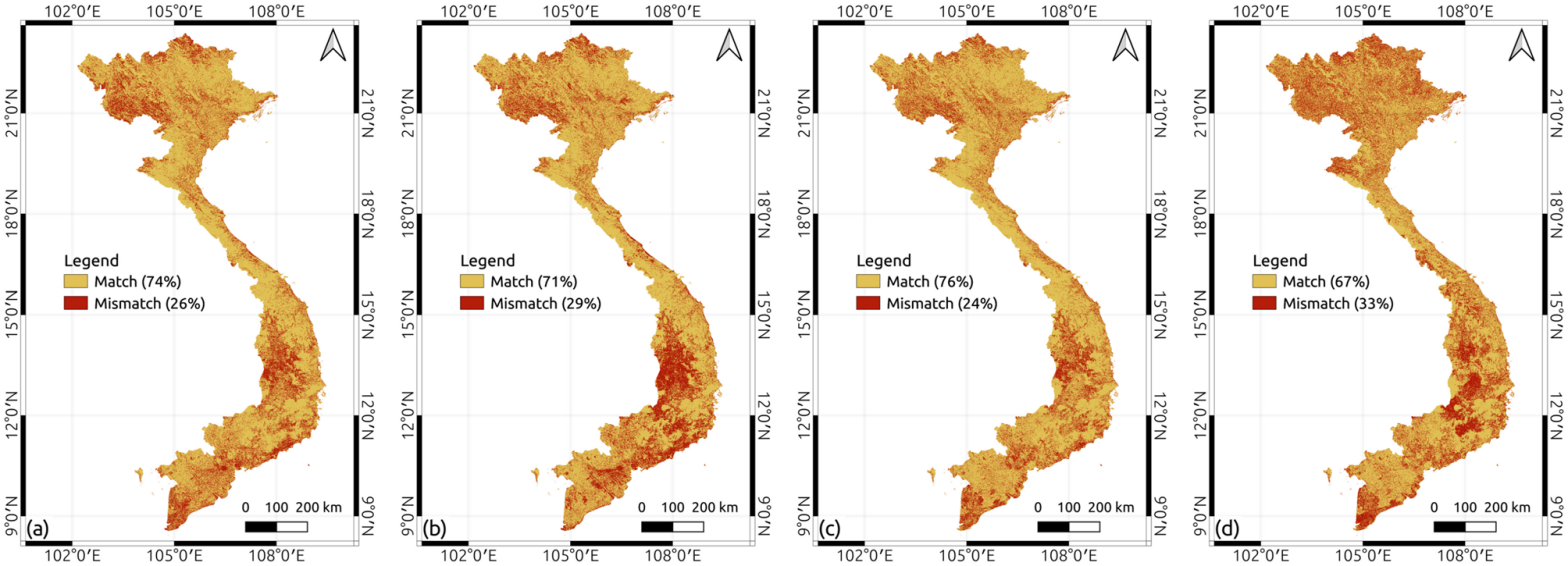
The pairwise comparison between our LULC map with other LULC maps by taking the difference between the maps. The red colors are matched pixels and the orange colors are mismatched pixels. (**a**) Our map versus DW, (**b**) Our map versus ESRI, (**c**) Our map versus ESA, (**d**) Our map versus JAXA’s 2016 map.

A pairwise comparison was conducted by measuring the difference between our map and the selected LULC maps. Two pixels of the same location were considered a match if they belonged to the same LULC category. The proportion of matched pixels represents the level of agreement between the two maps. Our map had the highest agreement level with ESA’s map, with 76% of matched pixels (see Fig. 6c), followed by 74% for DW (see Fig. 6a), 71% for ESRI (see Fig. 6b), and 67% for JAXA’s 2016 map (see Fig. 6d). By visual comparison, Fig. 6c presents slightly fewer red areas (mismatched pixels) than 6a, 6c, and 6d, especially for the northwest, the Central highland, and the southeast.

**Figure 7:**
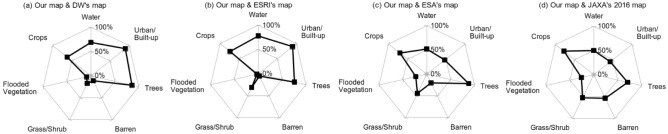
The percentage of matched pixels for each category in each pairwise comparison.

To evaluate category-level agreement, the number of matched pixels was calculated for each LULC category in each comparison case. The percentage of matched pixels for a category was computed by dividing the number of matched pixels by the total number of pixels for that category. The total number of pixels for each category was obtained from our LULC map. The level of agreement was categorized based on the percentage of matched pixels: high agreement ($\ge$ 60%), average agreement (30% - 60%), and low agreement ($\le$ 30%). Figure 7 presents spider charts, illustrating the percentage of matched pixels for each LULC category. The results revealed that the agreement was not equally distributed among the categories. Specifically, the categories of barren, grass/shrub, and flooded vegetation exhibited significantly lower agreement compared to the others. DW and ESRI demonstrated a strong agreement with our product for water, urban/built-up, tree, and crops but showed low agreement for barren, grass/shrub, and flooded vegetation (see Fig. 7a,b). In contrast, ESA showed a better agreement with our map for barren, grass/shrub, and flooded vegetation, but a lower agreement for water and urban/built-up (see Fig. 7c). Our map and JAXA’s 2016 map showed high agreement for crops and tree category, low agreement for flooded vegetation, and average agreement for the other categories (see Fig. 7d).

**Figure 8:**
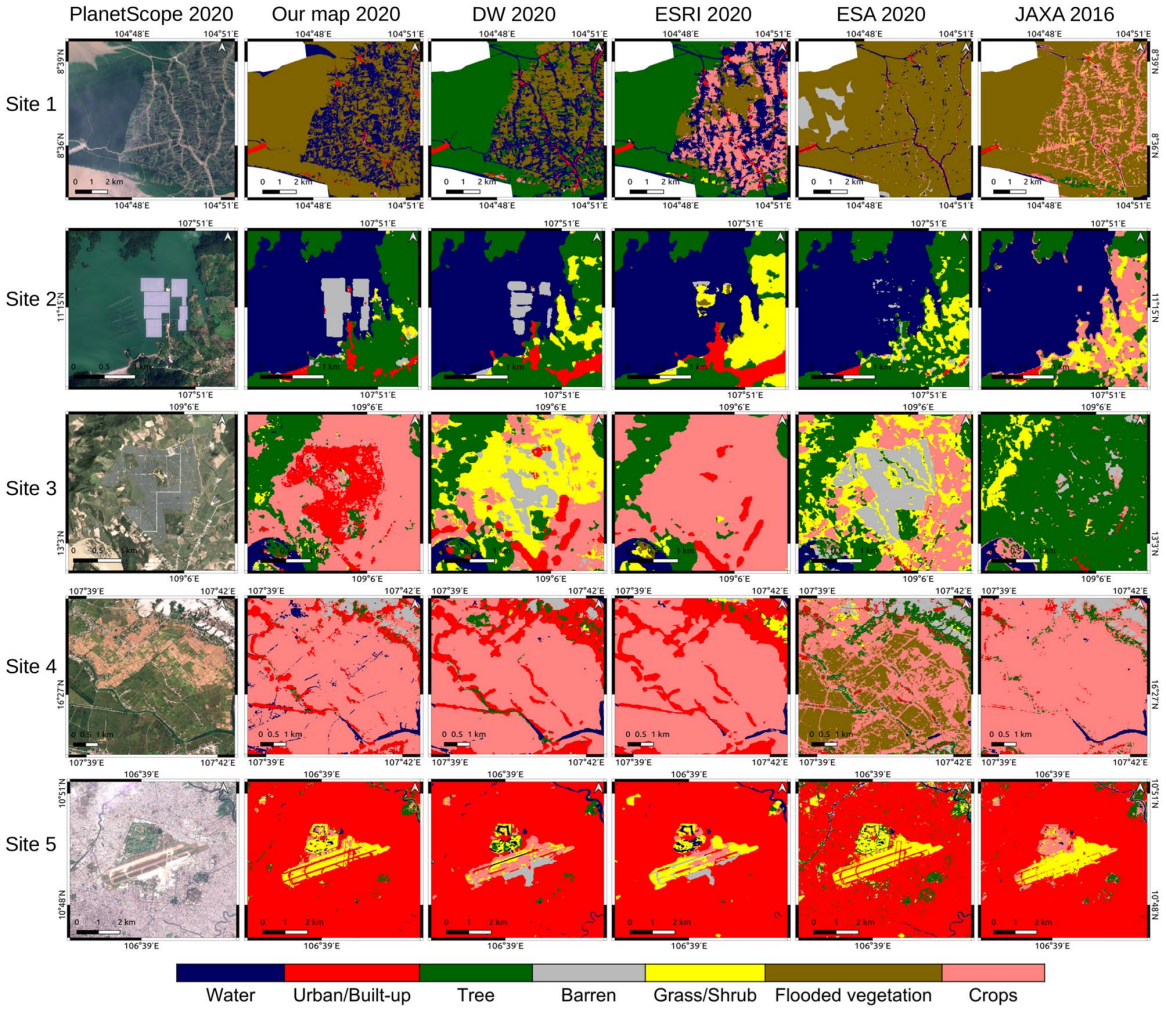
The visual comparison between our map and the other LULC maps in 5 sites, using PlanetScope’s images as a validation. Site 1: Comprising a mangrove forest on the left half and a combination of mangroves and water areas for aquatic farming on the right half. Site 2 and Site 3: Areas with solar panels in the center of the images. Site 4: An area encompassing paddy fields in the lower half and other crops in the upper half. Site 5: Tan Son Nhat Airport and its surrounding area in Ho Chi Minh City.

A visual comparison was conducted with PlanetScrope’s images at 5 sites (see Fig. 8). In Site 1, DW and ESRI misclassified dense mangrove forests as the tree category, ESA overestimated mangrove forests and JAXA’s 2016 map misclassified water as crops. Site 2 and Site 3 displayed areas with solar panels in the center of the images. While all maps misclassified the solar panels in Site 2, our map accurately detected the shape and border of the solar panels. In Site 3, our map correctly classified solar panels, whereas the other maps misclassified them into grass/shrub (DW), crops (ESRI), barren (ESA), and trees (JAXA’s 2016 map). In Site 4, all the maps well classified the crop areas except for ESA, which classified rice fields into flood vegetation, while DW and ESRI tend to overestimate urban/built-up areas. In Site 5, our map and ESA outperformed the others in classifying the runway of the airport and the grassland in the golf course near the airport. In general, our map has addressed limitations existing in the other LUC maps and achieved significantly higher accuracy than other LULC maps for Vietnam.

## Discussion

### Improvements of our LULC map

The produced 12-category LULC map has higher overall accuracy ($90.5\% \pm 1.2\%$) than the other 10-m LULC maps for Vietnam. Our previous study^49^ revealed that increasing the number of LULC categories by one could result in an approximate 1% decrease in overall accuracy. This decline in overall accuracy is attributed to the higher likelihood of misclassification within the expanded set of categories. Therefore, an overall accuracy of about 88% is considered a standard overall accuracy for a LULC map with 12 categories. Several factors contributed to the high accuracy of our map: (1) An extensive training dataset consisting of 121,521 points covers a diverse range of land cover conditions, supporting our CNN model in accurately detecting various landscapes; (2) The combination of optical and SAR images mitigated their own limitation, significantly enhancing the map’s accuracy; (3) The utilization of the time-feature CNN approach improved the capability of preserving spatial details in the output LULC map.

Among the global LULC products, ESA was better than the other global LULC maps in terms of pixel-level accuracy assessment. In addition, ESA’s map also exhibited better spatial detail than DW and ESRI, attributed to its use of the Random Forest classifier, whereas DW and ESRI relied on deep learning with spatial convolution. A visual comparison highlighted much salt/pepper noise in the ESA’s map and the clustered and generalized patterns^50^ in DW’s and ESRI’s map. Furthermore, several limitations were identified in the other LULC products as follows: (1) ESA’s map tend to misclassify paddy field into flooded vegetation such as herbaceous wetland; (2) DW and ESRI tend to overestimate urban/built-up areas; (3) JAXA’s map of Vietnam in 2016 misclassified aquatic farming into rice. In optical imagery, distinguishing impervious surfaces from bare soil proves a challenge due to their similar high spectral values, resulting in misclassifications. The misclassifications are evident in LULC products reliant solely on optical images, such as DW’s map and ESRI’s map (as seen in the top-right corner of Site 4 in Fig. 8). By integrating ALOS-2/PALSAR-2 images with Sentinel-2 images, our map effectively mitigates the confusion between barren and urban/built-up areas. This is attributed to the distinct backscattering characteristics of flat bare soil in PALSAR-2 images, which differ significantly from urban. However, it’s worth noting that challenges persist in regions where rough-surfaced bare soil may lead to misclassification with urban/built-up, as highlighted by Sun et al. (2019)^51^. The utilization of L-band ALOS-2/PALSAR-2 data offers the advantage of capturing information beneath dense vegetation canopies, enhancing the mapping accuracy of flooded vegetation^52^ . Additionally, C-band Sentinel-1 data proves advantageous for rice mapping^53^. As a result, the combination of L-band SAR and C-band SAR with optical imagery in our mapping methodology addresses and minimizes the misclassifications between rice paddies and flooded vegetation. This is a notable improvement over the ESA’s map, which used only C-band SAR and optical images (Site 4 in Fig. 8).

In terms of spatial detail, our map outperformed DW and ESRI in resolving the fine LULC features but with slightly less detail than ESA’s map. Two factors influenced the spatial detail of our map: (1) The use of PALSAR-2/ScanSAR and AW3D images with a spatial resolution of 25 m and 30 m, which limited the ability to reflect 10-m-spatial-resolution features in Sentinel-2 images onto final LULC map; (2) The process of converting data type from “float” to “byte” after the normalization, which could lead to loss of information due to the equalization of pixels with a small difference in their values. The second factor revolves around the trade-off between spatial detail and computational cost. When using data in “float” type, the information loss could be reduced, but at the expense of significantly increased computational demand. As a result, we must strike a balance between the level of detail in the output map and the computational cost that we are willing to incur.

Overall, the Hirayama’s time-feature convolution approach allows us to avoid the geospatial down-sampling, thus preserving the spatial resolution of the output to a great extent. This addresses the limitation of the conventional spatial convolution used in previous global LULC products while allowing the use of a standard CNN architecture. The time-feature CNN approach proved several advantages as follows: (1) Allowing the integration of data from multiple sources (satellite images, meteorological data, climate data, etc.) without creating sub-models for different data sources, resulting in a relatively simple model structure; (2) The ability to work with data from various temporal and spatial resolutions, providing flexibility in sensor imagery combination; (3) The robustness in detecting categories with significant seasonal differences, such as deciduous forests and seasonal crops, due to its design for time series data.

### The practicability of utilizing multi-year data for cloud-free optical image generation

In our study, we employed Sentinel-2 images from 2019, 2020, and 2021 to create median-composite images for the year 2020. Consequently, the output LULC map incorporated not only the LULC information of 2020 but also of 2019 and 2021 to some extent. While this approach may not be ideal for areas characterized by significant year-to-year changes in LULC, it is highly effective for places, where the LULC exhibits relatively small annual changes. Importantly, this approach stands out for its simplicity and ease of implementation when compared to complex methods used to recover missing data in optical imagery^54,55^. This advantage makes our approach readily applicable to missing data recovery on a large scale using Google Earth Engine.

### The demand for high-resolution LULC maps in Vietnam

In response to the 2030 Agenda and Sustainable Development Goals (SDGs), the Vietnamese government has implemented several key national strategies and action plans. These include the National Action Plan for the implementation of the 2030 sustainable development agenda^56^, the National Climate Change Strategy to 2050^57^, the National Action Programme on REDD+ by 2030^58^. Notably, the Vietnamese government has set ambitious targets for land use and the forestry sector, aiming to reduce greenhouse emissions by 70% and increase carbon absorption sinks by 20% by 2030. To achieve these SDGs, evaluating the current condition, status, and trend of national natural resources, including LULC at the national scale, is of utmost importance. For such context, high-resolution LULC maps play a critical role in providing necessary information for land use management, forest monitoring under the REDD+ program, and the conservation of natural ecosystems of the country.

### Potentials of time-feature CNN for future studies

**Figure 9:**
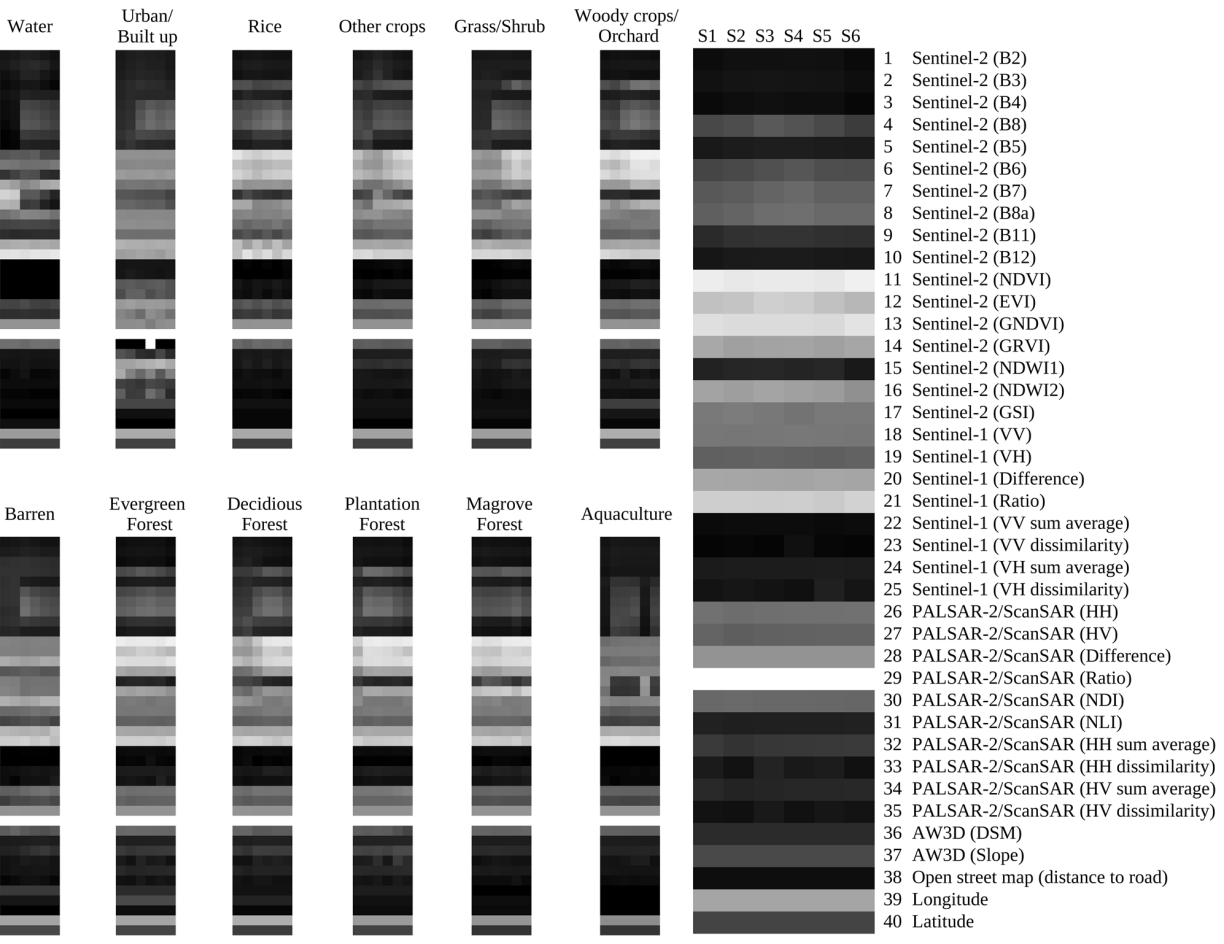
Examples of input data for the CNN model. The small images are 12 examples of training data for 12 LULC categories. The large image illustrates the structure of a training point with a horizontal axis (time axis, consisting of 6 seasons) and a vertical axis (feature axis, consisting of 40 features). This structure of input data is an extending version of the data structure proposed by Hirayama et al. (2022)^9^ (4 seasons and 30 features).

The time-feature CNN approach proposed by Hirayama et al. (2022)^9^ is different from the conventional CNNs by the capability to perform convolution across temporal and spectral domains, instead of spatial domain. Unlike spatial convolution, which works on group of spatially correlated pixels, the time-feature convolution approach necessitates the consideration of pixels in the time-series profile and the spectral profile. In the time-series profile, features were organized based on the continuity of the time sequence, spanning from Season 1 to Season 6 (Fig. 9). As for the spectral profile, our emphasis is placed on grouping features primarily by the sensor and secondarily by high-to-low spatial resolution. The spectral bands of Sentinel-2 were sequenced from short to long wavelength, aligning with the electromagnetic spectrum’s natural order. Although vegetation indices are independent, they were positioned after the spectral bands, reflecting their derivation from Sentinel-2’s bands. Following Sentinel-2, features from Sentinel-1, PALSAR-2/ScanSAR, AW3D, OpenStreet map, Longitude, and Latitude were incorporated. It is obvious that the continuity of spectral profile is obstructed by the independence of feature bands from different sensors. In addition, there is a dearth of prior studies addressing the optimal arrangement of the spectral profile for LULC classification. It is, therefore, strongly recommended that future studies explore the optimal organization of data from different sensors to improve the accuracy of LULC classification. On the contrary, an alternative approach can be to independently extract features from different sensors and then combine them. This approach may help reduce uncertainties linked to the arrangement of spectral profiles from different sensors.

Recently, the availability of high spatial resolution satellite sensors ($\le$ 10 m) has created numerous opportunities to acquire high-resolution time-series data on a large scale. The flexibility of utilizing time-series data from multiple sensors makes the application of the time-feature CNN for regional and global LULC mapping highly feasible. This approach has the potential to reduce discrepancies in global LULC maps when applying them to national and local scales, thanks to its ability to preserve geospatial patterns. Nevertheless, further research is needed to optimize the model’s structure and select optimal hyperparameters. Additionally, exploring the use of time-feature CNN with high-resolution images ($\le$ 3m) for seasonal vegetation monitoring is a promising research direction.

The coarse resolution image may influence the high spatial resolution image in presenting spatial details when they are combined. To address this, studies on feature-level fusion, result-level fusion, or combined models based on time-feature CNN should be conducted to figure out the suitable option for the combination of low-resolution and high-resolution images. Lastly, the time-feature CNN approach, which relies on point-based training data, offers the advantage of reducing the time required for training data collection, as these data can be shared with pixel-based machine learning studies.

## Conclusions

This study successfully generated a 10-m LULC map of Vietnam for the year 2020 with an impressive overall accuracy of $90.5\% \pm 1.2\%$, surpassing the accuracy of existing 10-m LULC maps for Vietnam. The produced map by the time-feature Convolutional Neural Network showed substantial enhancements compared to other global LULC maps, including: (1) The map’s spatial detail outperformed LULC products utilizing the spatial convolution approach such as DW’s and ESRI’s map; (2) Notable improvements were observed in addressing limitations present in other products, including the confusion of paddy fields with flooded vegetation in ESA’s map, the overestimation of urban/built-up areas in DW’s and ESRI’s maps, and the inaccurate classification of aquatic farming as rice fields in JAXA’s map; (3) The produced map stands out with 12 distinct categories, surpassing the 9 categories of Dynamic World, 11 categories of ESA World Cover, and 10 categories of ESRI Land Cover; (4) Our map offers a detailed classification legend for forest categories (evergreen, deciduous, plantation, and mangrove), a separate identification of rice and other crops, and a dedicated category for aquaculture.

This study proposes a straightforward and practical approach for constructing a time series of optical images spanning 6 seasons using data from multiple years. This method is easily implemented on Google Earth Engine and adaptable to various satellite images for data gap filling. Its simplicity is robust for a large-scale application. The time-feature CNN approach exhibits significant potential for producing high-accuracy LULC maps for cloudy regions by leveraging a combination of optical and SAR images. Given these achievements, further exploration of the proposed CNN approach is recommended, including the investigations of the model’s structure, the hyperparameter selection, and the feature arrangement to comprehensively understand its capabilities.

## Methods and materials

### Classification algorithm

In this section, this study provide a comprehensive description of the time-feature CNN approach originally developed by Hirayama et al. (2022)^9^. Unlike conventional CNNs that perform convolution on the geospatial domain, this approach was designed to operate on a time-feature domain. Consequently, the input data for the CNN model consists of not an array of pixels covering a geospatial area but an array of a time series of features (bands or indices) for a single pixel. Initially, this method was applied to create a LULC map of Japan using satellite data with 4 seasons. However, it is believed that increasing the number of seasons might help to provide useful information for the discrimination among the LULC categories. As a result, the modifications were made on the original source code of SACLASS2 software to enable the processing of satellite data within 6 seasons. Details of the method are described as follows:

Initially, the satellite images from different sensors underwent the re-projection to the Geographic Coordinate System. The integration of satellite data from different sensors was executed on the basis of a tile, covering an area of 1^o^x1^o^ (about 100 km^2^). Totally, 60 tiles are needed to fully cover area of Mainland Vietnam. This study employed GDAL (Geospatial Data Abstraction Library) and NumPy in Python environment to read the TIFF (Tagged Image File Format) files of satellite images and converted them into NumPy array. It is essential to highlight that our satellite data includes features (spectral bands, polarizations, vegetation indices, etc.) exhibiting different value range. Sentinel-2 bands, for instance, range from 0 to about 10,000, while values of vegetation indices are from -1 to 1. To ensure an equal contribution from all features to the classification result, a normalization step was applied. This entailed the conversion value of all features to [0, 1] range (see Equation 1). Following the normalization, features with a spatial resolution greater than 10 m were resampled to 10 m, employing the bilinear interpolation. Subsequently, for each 1^o^x1^o^ tile, all features for 6 seasons were combined and stored in the form of a NumPy array, referred to as a “combined image”.

Data in the combined image were arranged in a way that each pixel is considered as a function of *time* and *feature*. Here, *time* $\in$ {*S1, S2, S3, S4, S5, S6*} (see Table 5) and *feature* $\in$ {*B2, B3, ..., GSI, VH, VV, ..., Lat, Lon*}. Each pixel can be visualized as a two-dimensional grid, with a time axis on the horizontal dimension and a feature axis on the vertical dimension. Figure 9 shows examples of the grids in the form of greyscale color images. In the figure, there are 12 small images representing examples of training data for 12 LULC categories. The larger image is an enlarged view of one of the small images, providing a clearer depiction of the training data. It is worth noting that while a majority of features such as those from Sentinel-2, Sentinel-1, and PALSAR-2/ScanSAR exhibit seasonal variation, other features remain constant for all seasons. To preserve the local characteristics of the input data, latitude and longitude were added as features, positioning them at the edge of the image.

1 $$\begin{aligned} N_{b, i} = \frac{b_i - b_{min}}{b_{max} - b_{min}} \end{aligned}$$

where $N _{b,i}$ is the normalized value of pixel *i* of feature *b*, $b _{i}$ is the value of pixel *i* of feature *b* before normalization, $b _{min}$ is the minimum value of feature *b*, and $b _{max}$ is the maximum value of feature *b* over the study area.

After processing satellite data, raster images were formed from the training points. These training points were collected for each 1^o^x1^o^ tile and then exported to a text file. Each line in the text file contains data for a training point and includes a label of a category (numbered between 1 and 12 representing 12 LULC categories), latitude, and longitude information. To convert the latitude and longitude of a training point to indices of the corresponding row and column in a 1^o^x1^o^ raster, the methods in Equation 2 and Equation 3 were used.

2 $$\begin{aligned} x_{i} = \frac{lon_{i} - lon_{min}}{column_{tot}} \end{aligned}$$

3 $$\begin{aligned} y_{i} = \frac{lat_{i} - lat_{min}}{row_{tot}} \end{aligned}$$

where $x_{i}$ and $y_{i}$ are the corresponding column and row of the training point *i* in the 1^o^x1^o^ raster, $lon_{i}$ and $lat_{i}$ are the longitude and latitude of the training point *i*, $lon_{min}$ and $lat_{min}$ are the minimum longitude and minimum latitude of the $1^\text {o}$x$1^\text {o}$ raster, $column_{tot}$ and $row_{tot}$ are the total column and row of the 1^o^x1^o^ raster. In this study, each pixel has a size of 100 m^2^, which is equivalent to 8x$10^{-9}$ degree^2^. Within 1 degree^2^, there are a total of 11133^2^ pixels. With the row and column indices, the value of the pixel was determined based on the numeric label representing the corresponding LULC category. The raster of training points, referred to as a training image, contains pixels with data and no data. Hereinafter, training pixels were referred as the pixels containing valid data. At the position of each training pixel in the combined image, the data were collected. For each 1^o^x1^o^ tile, the data for all training pixels were collected and aggregated into a NumPy array. This Numpy array is the training dataset for the CNN model.

**Figure 10:**
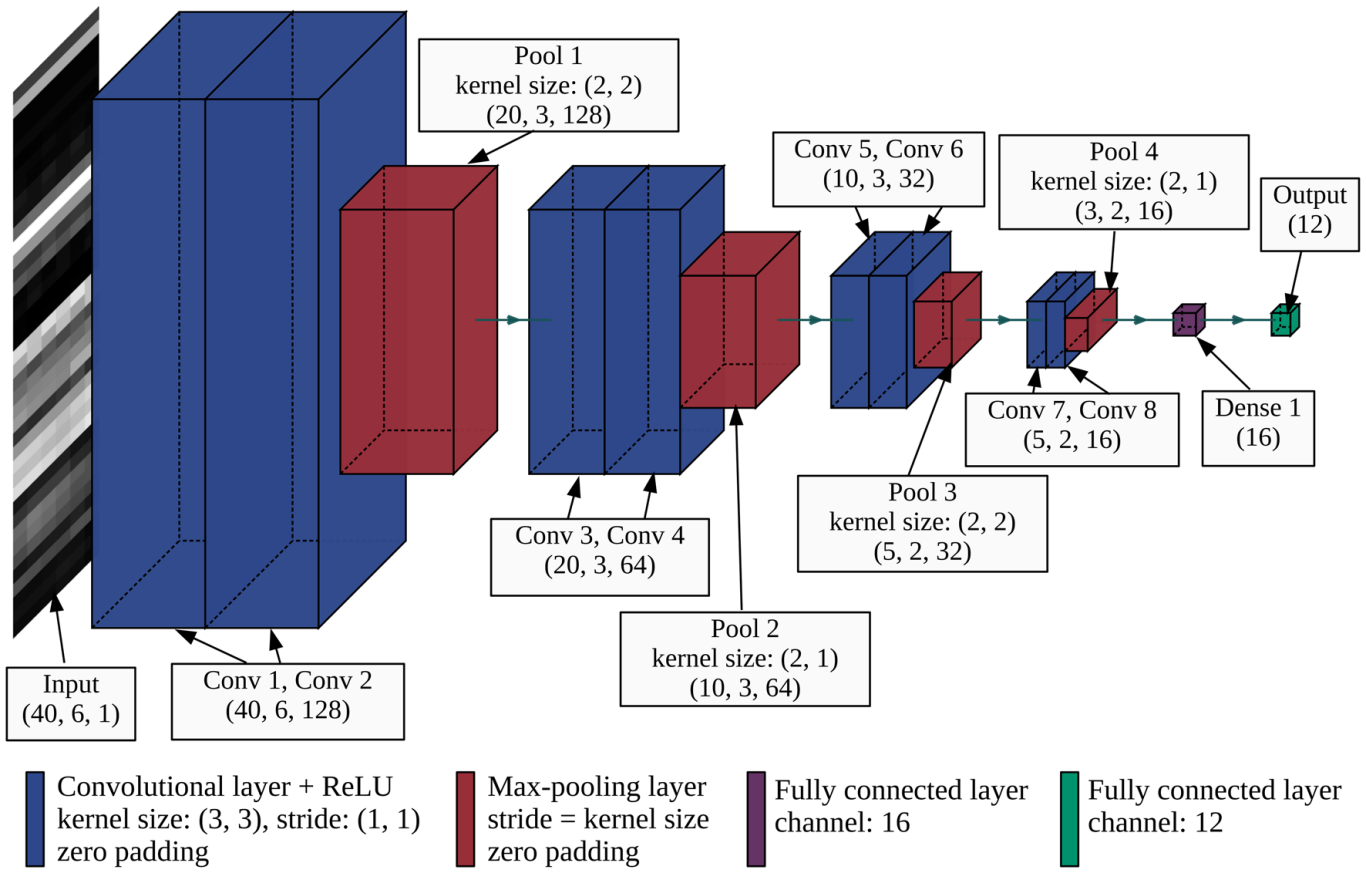
The structure of the CNN model used in this study by modifying the original CNN proposed by Hirayama et al. (2022)^9^. There are 4 pairs of convolutional layers and 4 max-pooling layers. The convolutional layers use ReLU as the activation function and the Dense 1 layer (fully connected layer) uses the softmax function.

The CNN architecture consists of a sequence of two convolution layers followed by one max pooling layer, as illustrated in Fig. 10. To prevent overfitting to the local characteristics of the input data, a dropout layer was applied before the fully connected layer. Activation functions were used as follows: ReLU for the convolution layers and softmax for the output layer. The loss function was multiclass tolerance entropy. To search for the optimal calculation condition, preliminary training process was performed on the model. In this process, 80% of the training data was used for training, while the remaining 20% served as the validation set. Based on the experimental results, Adam36 was selected as an optimization algorithm, with a batch size of 128. Early stopping was implemented with a patience parameter set to 10 epochs, and the learning reduction had a patience parameter of 2 epochs. The training process terminated after 23 epochs. Instead of employing data augmentation techniques, the complete set of 121,521 training points was used for the final learning of the model.

### Reference data collection and accuracy assessment

**Figure 11:**
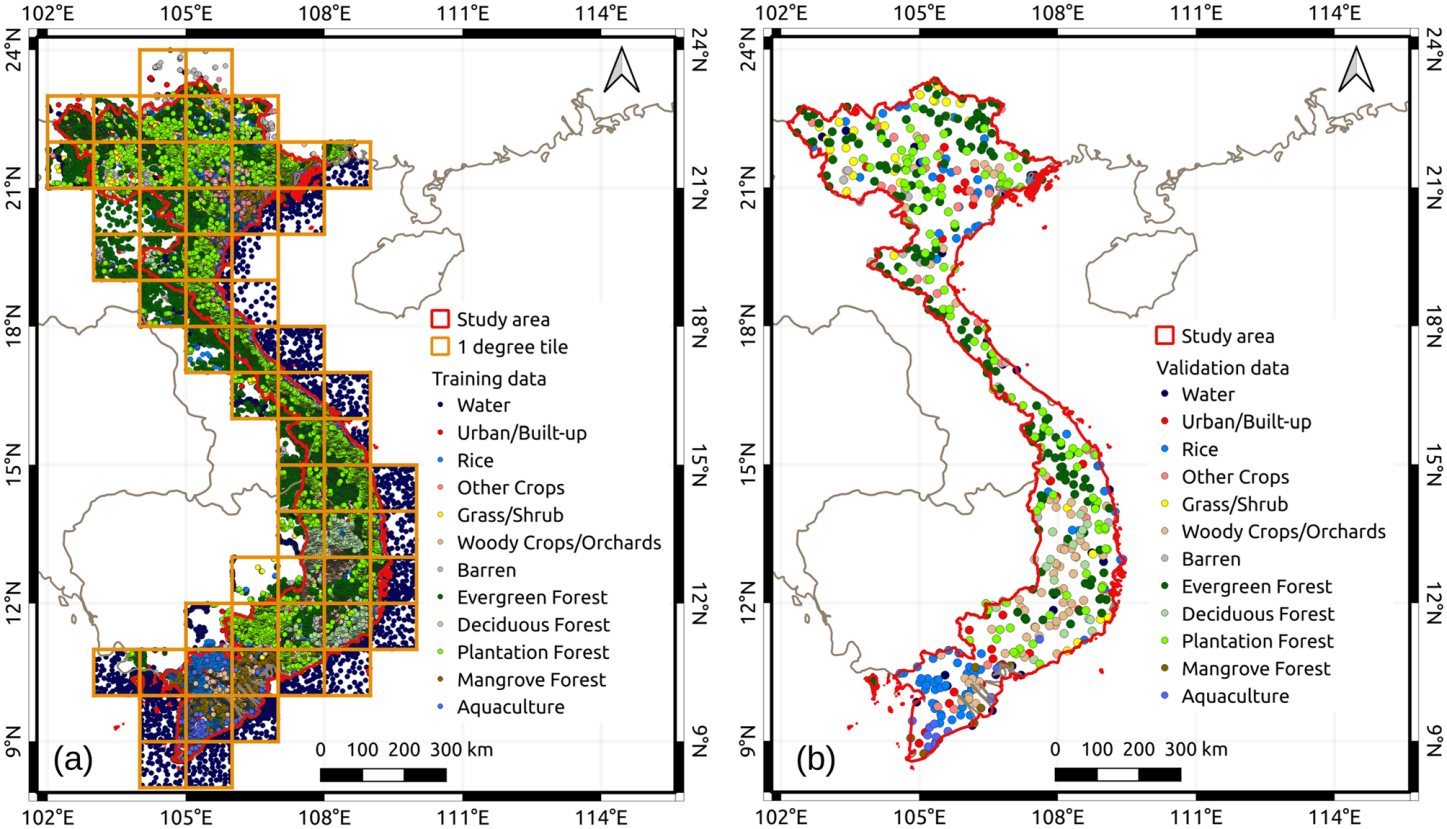
The distribution of reference data. The red polygons in both maps show the Mainland Vietnam. (**a**) The distribution of training data over the study area collected for each 1^o^x1^o^ tile. (**b**) The distribution of validation data collected by stratified random sampling.

To collect a large amount of training data, this study used the visual interpretation method on Sentinel-2 images, Google Earth Pro, Google Street View, and GPS photos taken from field surveys in 2018, 2019, 2020, and 2021. Our training data were collected in a point-based form and stored as vector data. Each training point represents a homogeneous LULC type with an area of 100 m^2^. To ensure a dense distribution of training data (see Fig. 11a), our team dedicated a tremendous effort to interpret about 171,000 samples for 12 LULC categories (see Table 2). 70% of the samples were used for the training process and the remaining 30% were used for preliminary accuracy assessment of the resulting map. The user’s accuracy derived from this preliminary accuracy assessment was used to calculate the sample size for random sampling of the validation data.

The validation dataset was collected separately from the training dataset using the stratified random sampling method. Our initial step involved estimating the sample size (*n*) using Equation 4, a method proposed by Cochran et al. (1977)^59^ and subsequently elaborated by Olofsson et al. (2014)^60^.

4 $$\begin{aligned} n = \frac{\bigg (\sum \limits _{i=1}^{k}\limits {W_{i}S_{i}}\bigg )^2}{[S(\hat{O})]^2 + (1/N)\sum \limits _{i=1}^{k}\limits {W_{i}S_{i}^2}} \approx \bigg (\frac{\sum \limits _{i=1}^{k}\limits {W_{i}S_{i}}}{S(\hat{O})}\bigg )^2 \end{aligned}$$

where *N* is the number of pixels in the LULC map used to create random samples, ${S(\hat{O})}$ is the desired standard error of the overall accuracy, $W_{i}$ is the area proportion of LULC category *i* on the map, $S_{i}$ is the standard deviation of the stratum *i*. $S_{i}$ was calculated based on the user’s accuracy ($U_{i}$) of the category *i* ($S_{i} = \sqrt{U_{i}(1 - U_{i})}$). Our objective is to achieve 1% for the standard error of the overall accuracy, resulting in a sample size of 583 (rounded up to 600). Using Qgis software, we conducted a random distribution process to distribute 600 points to the corresponding stratum of the LULC map (see Fig. 11b). The true LULC type of all random points were identified through the interpretation of Google Earth Pro, Sentinel-2, and Planet Scope’s images.

To evaluate the accuracy of the LULC map in a statistically robust manner, we followed a recommended methodology proposed by Olofsson et al. (2014). Firstly, an error matrix of sample count was constructed based on the validation data. Table 3 showed a generalized example of the error matrix of sample count for a LULC map with k categories. Secondly, an estimated error matrix (see Table 4) was created by multiplying each row of Table 3 by the area proportion of the category in that row. The estimated area proportion in cell i,j of Table 4 was calculated using Equation 5.

5 $$\begin{aligned} \hat{p}_{ij} = W_{i}\frac{n_{ij}}{n_{i\cdot }} \end{aligned}$$

where $W_{i}$ is the area proportion of the category *i* on the map ($W_{i} = {A_{m,i}} \div {A_{tot}}$, in which $A_{m,i}$ is mapped area of category *i* and $A_{tot}$ is the total area of the map), $n_{ij}$ and $n_{i\cdot }$ are from Table 3. The user’s accuracy ($\hat{U}_{i}$), the producer’s accuracy ($\hat{P}_{i}$) of category *i*, and overall accuracy ($\hat{O}$) are as follows:

6 $$\begin{aligned} \hat{U}_{i} = \frac{\hat{p}_{ii}}{\hat{p}_{i\cdot }} \end{aligned}$$

7 $$\begin{aligned} \hat{P}_{i} = \frac{\hat{p}_{ii}}{\hat{p}_{\cdot {i}}} \end{aligned}$$

8 $$\begin{aligned} \hat{O} = \sum \limits _{i=1}^{k}\limits {p_{ii}} \end{aligned}$$

The estimated variance of the overall accuracy [$\hat{V}(\hat{O})$], user’s accuracy [$\hat{V}(\hat{U_{i}})$], and producer’s accuracy [$\hat{V}(\hat{P_{i}})$] of category *i* are as follows:

9 $$\begin{aligned}{} & {} \hat{V}(\hat{O}) = \sum \limits _{i=1}^{k}\limits {W_{i}^2\hat{U_{i}}(1-\hat{U_{i}})/(n_{i\cdot }-1)} \end{aligned}$$

10 $$\begin{aligned}{} & {} \hat{V}(\hat{U_{i}}) = \hat{U_{i}}(1-\hat{U_{i}})/(n_{i\cdot }-1) \end{aligned}$$

11 $$\begin{aligned}{} & {} \hat{V}(\hat{P_{i}}) = \frac{1}{(\hat{p}_{\cdot {i}})^2}\bigg [W_{i}^2(1-\hat{P}_{i})^2\frac{\hat{U}_{i}(1-\hat{U}_{i})}{n_{i\cdot }-1} + \hat{P}_{i}^2\sum \limits _{j\ne i}^{k}\limits {W_{j}^2\frac{n_{ji}}{n_{j\cdot }}(1-\frac{n_{ji}}{n_{j\cdot }})/(n_{j\cdot }-1)}\bigg ] \end{aligned}$$

It is noteworthy that there is no $W_{i}^2\frac{n_{ii}}{n_{i\cdot }}(1-\frac{n_{ii}}{n_{i\cdot }})/(n_{i\cdot }-1)$ in the second term of Equation 12, because of $j \ne i$

**Table 2:** Definition of LULC categories, number of training and validation data.

**ID**	**Category**	**Definition**	**Number of training points**	**Number of validation points**
1	Water	Open water bodies (e.g. rivers and oceans) and closed water bodies that are enclosed by land or other artificial barriers (e.g. lakes, ponds, and reservoirs).	10,643	24
2	Urban/built-up	Impervious surface and man-made structures (e.g. buildings, houses, paved roads, greenhouses, and solar panels), excluding farm roads, irrigation roads, and trails.	17,078	30
3	Rice	Rice fields that are single season or multiple seasons. Terrace rice fields are also included.	22,111	72
4	Other Crops	Crops other than rice and woody crops (e.g. corn, bean, and sugar cane)	6,634	25
5	Grass/Shrub	Land with non-tree herbaceous vegetation, grasslands, spare shrub (less than 5m in height and greater than 10% canopy cover), golf courses, etc.	2,641	48
6	Woody Crops/Orchards	Perennial woody crops (e.g. coffee, tea, cashew) and woody orchards (e.g. mango, litchi, and dragon fruit)	19,340	78
7	Barren	Lands with less than 10% of vegetation such as sandy beaches, sand dunes, bare rocks, bare soil, playgrounds	3,572	18
8	Evergreen Forest	Lands dominated by natural evergreen trees with > 60% coverage and > 5m in height	19,435	161
9	Deciduous Forest	Lands dominated by deciduous trees with > 60% coverage and > 5m in height	2,521	20
10	Plantation Forest	Lands dominated by plantation trees with > 60% coverage and > 5m in height	12,618	100
11	Mangrove Forest	Lands dominated by mangrove trees with > 60% coverage and > 5m in height	3,442	10
12	Aquaculture	Lands that are permanently or temporarily flooded used for aquatic farming such as fish ponds and shrimp ponds	4,486	14

**Table 3:** An error matrix of sample count for LULC map with k categories. Map categories are rows and reference categories are columns.

**Class**	**1**	**2**	$\cdots$	**i**	**j**	$\cdots$	**k**	**Total**
**1**	$n_{11}$	$n_{12}$	$\cdots$	$n_{1i}$	$n_{1j}$	$\cdots$	$n_{1k}$	$n_{1\cdot }$
**2**	$n_{21}$	$n_{22}$	$\cdots$	$n_{2i}$	$n_{2j}$	$\cdots$	$n_{2k}$	$n_{2\cdot }$
$\vdots$	$\vdots$	$\vdots$	$\ddots$	$\cdots$	$\cdots$	$\ddots$	$\cdots$	$\cdots$
**i**	$n_{i1}$	$n_{i2}$	$\cdots$	$n_{ii}$	$n_{ij}$	$\cdots$	$n_{ik}$	$n_{i\cdot }$
**j**	$n_{j1}$	$n_{j2}$	$\cdots$	$n_{ji}$	$n_{jj}$	$\cdots$	$n_{jk}$	$n_{j\cdot }$
$\vdots$	$\vdots$	$\vdots$	$\ddots$	$\cdots$	$\cdots$	$\ddots$	$\cdots$	$\cdots$
**k**	$n_{k1}$	$n_{k2}$	$\cdots$	$n_{ki}$	$n_{kj}$	$\cdots$	$n_{kk}$	$n_{k\cdot }$
**Total**	$n_{\cdot 1}$	$n_{\cdot 2}$	$\cdots$	$n_{\cdot {i}}$	$n_{\cdot {j}}$	$\cdots$	$n_{\cdot {k}}$	*n*

**Table 4:** An error matrix of estimated area proportion. Map categories are rows and reference categories are columns.

**Class**	**1**	**2**	$\cdots$	**i**	**j**	$\cdots$	**k**	**Total**
**1**	$\hat{p}_{11}$	$\hat{p}_{12}$	$\cdots$	$\hat{p}_{1i}$	$\hat{p}_{1j}$	$\cdots$	$\hat{p}_{1k}$	$\hat{p}_{1\cdot }$
**2**	$\hat{p}_{21}$	$\hat{p}_{22}$	$\cdots$	$\hat{p}_{2i}$	$\hat{p}_{2j}$	$\cdots$	$\hat{p}_{2k}$	$\hat{p}_{2\cdot }$
$\vdots$	$\vdots$	$\vdots$	$\ddots$	$\cdots$	$\cdots$	$\ddots$	$\cdots$	$\cdots$
**i**	$\hat{p}_{i1}$	$\hat{p}_{i2}$	$\cdots$	$\hat{p}_{ii}$	$\hat{p}_{ij}$	$\cdots$	$\hat{p}_{ik}$	$\hat{p}_{i\cdot }$
**j**	$\hat{p}_{j1}$	$\hat{p}_{j2}$	$\cdots$	$\hat{p}_{ji}$	$\hat{p}_{jj}$	$\cdots$	$\hat{p}_{jk}$	$\hat{p}_{j\cdot }$
$\vdots$	$\vdots$	$\vdots$	$\ddots$	$\cdots$	$\cdots$	$\ddots$	$\cdots$	$\cdots$
**k**	$\hat{p}_{k1}$	$\hat{p}_{k2}$	$\cdots$	$\hat{p}_{ki}$	$\hat{p}_{kj}$	$\cdots$	$\hat{p}_{kk}$	$\hat{p}_{k\cdot }$
**Total**	$\hat{p}_{\cdot 1}$	$\hat{p}_{\cdot 2}$	$\cdots$	$\hat{p}_{\cdot {i}}$	$\hat{p}_{\cdot {j}}$	$\cdots$	$\hat{p}_{\cdot {k}}$	1

### Satellite images pre-processing

This study used satellite images from multiple sensors, including Sentinel-2, Sentinel-1, ALOS-2/PALSAR-2, ALOS/PRISM, and OpenStreetMap data. To capture seasonal variations, a median-composite image for each two-month period was created, except for ALOS/PRISM and OpenStreetMap data. Table 5 described details of input satellite data used in this study.

**Table 5:** Satellite images and auxiliary data used in this study.

**Data**	**Processing level**	**Band/Polarization**	**Spatial resolution**	**Time/Seasons**
Sentinel-2	Level 2A	B2, B3, B4, B5, B6, B7, B8, B8A, B11, B12, NDVI, EVI, GNDVI, GRVI, NDWI1, NDWI2, GSI	10 m, 20 m	S1: 2020/01/01-2020/02/29, S2: 2020/03/01-2020/04/30, S3: 2020/05/01-2020/06/30, S4: 2020/07/01-2020/08/31, S5: 2020/09/01-2020/10/31, S6: 2020/11/01-2020/12/31.
Sentinel-1	Level 1.0	VH, VV, VH-VV, VH/VV, VH_avg, VV_avg, VH_diss, VV_diss	10 m	
ALOS-2/PALSAR-2/ScanSAR	Level 2.2	HH, HV, HH-HV, HH/HV, NDI, NLI, HH_avg, HV_avg, HH_diss, HV_diss	25 m	
ALOS/PRISM/DSM (AW3D30)	Version 2.1	DSM, Slope	30 m	Between 2006 and 2011
Road networks	-	Distance to road	-	2020

#### Sentinel-2

Sentinel-2 Level-2A (L2A) dataset^46^ provides radiometrically calibrated surface reflectance images, which is an advanced processing level from the Level-1C using Sen2Cor software^61^. Our study utilized both 10-m bands (B2, B3, B4, and B8) and 20-m bands (B5, B6, B7, B8A, B11, B12). We selected images with less than 30% of cloud to preliminary filter heavy cloud-contaminated images. After that, the cloud and its shadow were masked using a combination of Sentinel-2 Cloud Probability (s2cloudless), Cloud Displacement Index (CDI), and Directional Distance Transform (DDT). This cloud masking technique was applied to extensively mask cloud and cloud shadow of L2A images^5^.

During the investigation of Sentinel-2 images for Mainland Vietnam, the heavily cloud-contaminated images occurred from May to October, resulting in large areas of missing data for images in these months. To fill data gaps caused by the cloud and eliminate bad pixels, this study applied a simple and practical approach using a time series of multiple years as follows:

Firstly, monthly median-composite images were created for the years 2019, 2020, and 2021. Subsequently, median-composite images of 6 seasons were generated using the previously derived monthly images (see Equation 12).

12 $$\begin{aligned} \begin{aligned} Img\_t\_{\text {t+1}\_20} = Median({Img\_t\_20}, {Img\_t\_20},\\ {Img\_\text {t+1}\_20}, {Img\_\text {t+1}\_20},\\ {Img\_\text {t-1}\_20}, {Img\_\text {t+2}\_20},\\ {Img\_t\_19}, {Img\_\text {t+1}\_19},\\ {Img\_t\_21}, {Img\_\text {t+1}\_21}) \end{aligned} \end{aligned}$$

where:

*t* $$\in$$ {1,3,5,7,9,11},

*Img_t_*t+1*_20* is the median-composite image between time *t* and time t+1 in 2020,

*Median()* refers to the median method for image collection in Google Earth Engine,

*Img_*t-1*_20*, *Img_t_20*, *Img_*t+1*_20*, and *Img_*t+2*_20* are the median-composite images for time t-1, *t*, t+1, and t+2 in 2020, respectively,

*Img_t_19* and *Img_*t+1*_19* are the median-composite images for time *t* and time t+1 in 2019, respectively,

*Img_t_21* and *Img_*t+1*_21* are the median-composite images for time *t* and time t+1 in 2021, respectively.

In Equation 12, we doubled *Img_t_20* and *Img_*t+1*_20* for the sake of increasing their weights. Because our strategy is to ensure a major contribution of data in time *t* and *t+1* to the resulting median-composite image.

Secondly, in the cases of $t=1$ (January) and $t=11$ (November), the *Img_*t-1*_20* and *Img_*t+2*_20* were removed, respectively. Because there are no such images for these seasons in 2020. In case $t=5$, $t=7$, and $t=9$, we added *Img_*t-1*_19*, *Img_*t+2*_19*, *Img_*t-1*_21*, and *Img_*t+2*_21* to Equation 12. Because large areas of missing data for those seasons required more images to fill the data gap.

The pre-processing for Sentinel-2 was implemented on Google Earth Engine, and the resulting images were downloaded to our local computer. To increase the number of feature bands, several vegetation indices were used (see from Equation 13 to 19), including Normalized Difference Vegetation Index (NDVI), Enhanced Vegetation Index (EVI), Green Normalized Difference Vegetation Index (GNDVI), Green-Red Vegetation Index (GRVI), Normalized Difference Water Index 1 (NDWI1), Normalized Difference Water Index 2 (NDWI2), Topsoil Grain Size Index (GSI).

13 $$\begin{aligned}{} & {} NDVI = \frac{NIR - RED}{NIR + RED} \end{aligned}$$

14 $$\begin{aligned}{} & {} EVI = \frac{2.5(NIR - RED)}{NIR + 6RED - 7.5BLUE + 1} \end{aligned}$$

15 $$\begin{aligned}{} & {} GNDVI = \frac{NIR - GREEN}{NIR + GREEN} \end{aligned}$$

16 $$\begin{aligned}{} & {} GRVI = \frac{GREEN - RED}{GREEN + RED} \end{aligned}$$

17 $$\begin{aligned}{} & {} NDWI1 = \frac{RED - SWIR1}{RED + SWIR1} \end{aligned}$$

18 $$\begin{aligned}{} & {} NDWI2 = \frac{NIR - SWIR1}{NIR + SWIR1} \end{aligned}$$

19 $$\begin{aligned}{} & {} GSI = \frac{RED - BLUE}{RED + GREEN + BLUE} \end{aligned}$$

#### Sentinel-1

This study used the Sentinel-1 GRD (Ground Range Detected) product on Google Earth Engine^62^. The GRD product provides calibrated and ortho-corrected images of 10-m C-band SAR (Synthetic Aperture Radar), including thermal noise removal, and radiometric calibration with pixels containing $\sigma ^0$ values in the Decibel unit. For slope correction, the angular-based radiometric slope correction method^63^ was applied using the default model for vegetation covers and the Digital Elevation Model (DEM) from the Shuttle Radar Topography Mission product. To reduce the speckle noises, the Refined Lee Filter method was applied with a 3x3 moving window. The javascript code for the speckle filter was acquired from the supplementary material of Hird et al. (2017)^64^. Dual-band cross-polarization (VV and VH) were used for the calculation of the *Difference* and *Ratio* indices (see Equations 20 and 21). Furthermore, we incorporated two texture metrics for each polarization, specifically *Sum Average* and *Dissimilarity*, using the “ee.Image.glcTexture()” function in Google Earth Engine. The processed images have 8 bands, including $\gamma _{VH}^0$, $\gamma _{VV}^0$, *Difference*, *Ratio*, and 4 textured bands.

20 $$\begin{aligned}{} & {} Difference = \gamma _{VH}^0 - \gamma _{VV}^0 \end{aligned}$$

21 $$\begin{aligned}{} & {} Ratio = \frac{\gamma _{VH}^0}{\gamma _{VV}^0} \end{aligned}$$

#### ALOS-2/PALSAR-2/ScanSAR data

The PALSAR/PALSAR-2 data of JAXA have been the most frequently used L-band SAR for forest mapping and coastal area change analysis^65^. Because of the ability of L-band microwaves to penetrate through dense vegetation, it has a good capability to detect dense forests and flooded vegetation such as mangrove forests. This study used 25-m PALSAR-2/ScanSAR level 2.2 images provided by JAXA via Google Earth Engine^66^. The level 2.2 product provides ortho-rectified and slop-corrected images using ALOS World 3D (AW3D) data. To remove the speckle noise, we applied a Refined Lee Filter with a 5x5 moving window and converted the pixel value from digital number (DN) to gamma-naught (unit in decibel) using Equation 22, in which *CF* is the calibration factor with the value of -83 dB^67^. HH and HV polarizations were used for creating radar indices, including *Difference* (see Equation 23), *Ratio* (see Equation 24), Normalized Difference Index (*NDI*) (see Equation 25), *NLI* index (see Equation 26)^41^, and two textured metrics (*Sum Average* and *Dissimilarity*) for each polarization. The processed image contains 10 bands, including $\gamma _{HH}^0$, $\gamma _{HH}^0$, *Difference*, *Ratio*, *NDI*, *NLI*, and 4 textured bands.

22 $$\begin{aligned}{} & {} \gamma ^0 = 10 \times log_{10}<DN^2> + CF \end{aligned}$$

23 $$\begin{aligned}{} & {} Difference = \gamma _{HH}^0 - \gamma _{HV}^0 \end{aligned}$$

24 $$\begin{aligned}{} & {} Ratio = \frac{\gamma _{HH}^0}{\gamma _{HV}^0} \end{aligned}$$

25 $$\begin{aligned}{} & {} NDI = \frac{\gamma _{HH}^0 - \gamma _{HV}^0}{\gamma _{HH}^0 + \gamma _{HV}^0} \end{aligned}$$

26 $$\begin{aligned}{} & {} NLI = \frac{\gamma _{HH}^0 \times \gamma _{HV}^0}{\gamma _{HH}^0 + \gamma _{HV}^0} \end{aligned}$$

#### ALOS World 3D and OpenStreetMap data

Auxiliary data such as digital surface model (DSM) and OpenStreetMap are popularly used to improve the accuracy of land use land cover classification^68–71^. This study used altitude and slope derived from ALOS World 3D (AW3D) with 30 m spatial resolution^72^. Regarding OpenStreetMap data, vector data of road networks for 2020 were used, containing information on primary roads, secondary roads, and residential roads^73^. A distance to road map was derived from the raster map of the road network, which was the result of the conversion process from vector to raster on QGIS software.

### Supplementary Information

Supplementary Information.

## Data Availability

The 10-m LULC map of 2020 for Vietnam is available on the website of the Earth Observation Research Center (EORC) of JAXA. Users can download the map by accessing the following link: https://www.eorc.jaxa.jp/ALOS/en/dataset/lulc/lulc_vnm_v2309_e.htm. The thumbnail views of Sentinel-2 RGB images can be found at the following link: https://pen.envr.tsukuba.ac.jp/~phantom/S2_6seasons/. Due to the large file size of Sentinel-2 images, users who want to obtain 6 seasons of Sentinel-2 for Vietnam can directly send the request to the corresponding author. The satellite data can be shared after considering the user’s requests. In addition, the reference data can be shared upon a reasonable request.

## References

[1] Feddema JJ (2005). The importance of land-cover change in simulating future climates. Science.

[2] Naikoo MW, Rihan M, Ishtiaque M (2020). Analyses of land use land cover (lulc) change and built-up expansion in the suburb of a metropolitan city: Spatio-temporal analysis of delhi ncr using landsat datasets. J. Urban Manag..

[3] Karakuş CB (2019). The impact of land use/land cover (lulc) changes on land surface temperature in sivas city center and its surroundings and assessment of urban heat island. Asia-Pac. J. Atmos. Sci..

[4] Glade T (2003). Landslide occurrence as a response to land use change: a review of evidence from new zealand. CATENA.

[5] Brown CF (2022). Dynamic world, near real-time global 10 m land use land cover mapping. Sci. Data.

[6] Zanaga, D. *et al.* Esa worldcover 10 m 2020 v100. zenodo (2021).

[7] Karra, K. *et al.* Global land use/land cover with sentinel 2 and deep learning. In *2021 IEEE international geoscience and remote sensing symposium IGARSS*, 4704–4707 (IEEE, 2021).

[8] Takahashi, M. *et al.* Jaxa high resolution land-use and land-cover map of japan. In *2013 IEEE International Geoscience and Remote Sensing Symposium-IGARSS*, 2384–2387 (IEEE, 2013).

[9] Hirayama, S. *et al.* Generation of high-resolution land use and land cover maps in japan version 21.11. *J. Remote Sens. Soc. Japan* **42**, 199–216 (2022).

[10] Xu Y (2020). Annual 30-m land use/land cover maps of china for 1980–2015 from the integration of avhrr, modis and landsat data using the bfast algorithm. Sci. China Earth Sci..

[11] Mirmazloumi SM (2022). Elulc-10, a 10 m european land use and land cover map using sentinel and landsat data in google earth engine. Remote Sensing.

[12] Asenso Barnieh B, Jia L, Menenti M, Zhou J, Zeng Y (2020). Mapping land use land cover transitions at different spatiotemporal scales in west africa. Sustainability.

[13] Digra M, Dhir R, Sharma N (2022). Land use land cover classification of remote sensing images based on the deep learning approaches: a statistical analysis and review. Arab. J. Geosci..

[14] Kattenborn T, Leitloff J, Schiefer F, Hinz S (2021). Review on convolutional neural networks (cnn) in vegetation remote sensing. ISPRS J. Photogramm. Remote. Sens..

[15] Moharram, M. A. & Sundaram, D. M. Land use and land cover classification with hyperspectral data: A comprehensive review of methods, challenges and future directions. *Neurocomputing* (2023).

[16] Campos-Taberner M (2020). Understanding deep learning in land use classification based on sentinel-2 time series. Sci. Rep..

[17] Ma L (2019). Deep learning in remote sensing applications: A meta-analysis and review. ISPRS J. Photogramm. Remote. Sens..

[18] Maggiori E, Tarabalka Y, Charpiat G, Alliez P (2016). Convolutional neural networks for large-scale remote-sensing image classification. IEEE Trans. Geosci. Remote Sens..

[19] LeCun Y, Bengio Y, Hinton G (2015). Deep learning. nature.

[20] Mohammadi M, Sharifi A (2021). Evaluation of convolutional neural networks for urban mapping using satellite images. J. Indian Soc. Remote Sens..

[21] Saralioglu E, Gungor O (2022). Semantic segmentation of land cover from high resolution multispectral satellite images by spectral-spatial convolutional neural network. Geocarto Int..

[22] Zhang C, Wei S, Ji S, Lu M (2019). Detecting large-scale urban land cover changes from very high resolution remote sensing images using cnn-based classification. ISPRS Int. J. Geo Inf..

[23] Zhang M, Lin H, Wang G, Sun H, Fu J (2018). Mapping paddy rice using a convolutional neural network (cnn) with landsat 8 datasets in the dongting lake area, china. Remote Sensing.

[24] Ali K, Johnson BA (2022). Land-use and land-cover classification in semi-arid areas from medium-resolution remote-sensing imagery: A deep learning approach. Sensors.

[25] Zaabar N, Niculescu S, Kamel MM (2022). Application of convolutional neural networks with object-based image analysis for land cover and land use mapping in coastal areas: A case study in ain témouchent, algeria. IEEE J. Sel. Top. Appl. Earth Obs. Remote Sens..

[26] Bhosle K, Musande V (2019). Evaluation of deep learning cnn model for land use land cover classification and crop identification using hyperspectral remote sensing images. J. Indian Soc. Remote Sens..

[27] Guidici D, Clark ML (2017). One-dimensional convolutional neural network land-cover classification of multi-seasonal hyperspectral imagery in the san francisco bay area, california. Remote Sens..

[28] Hasan, H., Shafri, H. Z. & Habshi, M. A comparison between support vector machine (svm) and convolutional neural network (cnn) models for hyperspectral image classification. In *IOP Conference Series: Earth and Environmental Science*, vol. 357, 012035 (IOP Publishing, 2019).

[29] Ienco D, Gaetano R, Dupaquier C, Maurel P (2017). Land cover classification via multitemporal spatial data by deep recurrent neural networks. IEEE Geosci. Remote Sens. Lett..

[30] Benedetti P (2018). A deep learning architecture for multiscale multimodal multitemporal satellite data fusion. IEEE J. Sel. Top. Appl. Earth Obs. Remote Sens..

[31] Dou P, Shen H, Li Z, Guan X (2021). Time series remote sensing image classification framework using combination of deep learning and multiple classifiers system. Int. J. Appl. Earth Obs. Geoinf..

[32] Ienco D, Interdonato R, Gaetano R, Minh DHT (2019). Combining sentinel-1 and sentinel-2 satellite image time series for land cover mapping via a multi-source deep learning architecture. ISPRS J. Photogramm. Remote. Sens..

[33] Song H, Liu Q, Wang G, Hang R, Huang B (2018). Spatiotemporal satellite image fusion using deep convolutional neural networks. IEEE J. Sel. Top. Appl. Earth Obs. Remote Sens..

[34] Springenberg, J. T., Dosovitskiy, A., Brox, T. & Riedmiller, M. Striving for simplicity: The all convolutional net. arXiv preprint arXiv:1412.6806 (2014).

[35] Yu, F. & Koltun, V. Multi-scale context aggregation by dilated convolutions. arXiv preprint arXiv:1511.07122 (2015).

[36] Zeiler, M. D., Krishnan, D., Taylor, G. W. & Fergus, R. Deconvolutional networks. In *2010 IEEE Computer Society Conference on computer vision and pattern recognition*, 2528–2535 (IEEE, 2010).

[37] Thuaire, B. *et al.* Assessing the biodiversity in viet nam: Analysis of impacts from economic sectors. Tech. Rep., World Wide Fund for Nature (WWF-Vietnam) (2021). https://www.biodev2030.org/wp-content/uploads/2022/01/Final-Report_Biodiversity-assessment-in-Vietnam-Analysis-of-impact-of-economic-sectors-ENG.pdf [Accessed: August 25, 2023].

[38] Nguyen M-H, Jones TE (2022). Predictors of support for biodiversity loss countermeasure and bushmeat consumption among vietnamese urban residents. Conserv. Sci. Pr..

[39] Shahbaz M, Haouas I, Van Hoang TH (2019). Economic growth and environmental degradation in vietnam: is the environmental kuznets curve a complete picture?. Emerg. Mark. Rev..

[40] Truong VT (2019). Jaxa annual forest cover maps for vietnam during 2015–2018 using alos-2/palsar-2 and auxiliary data. Remote Sensing.

[41] Hoang TT, Truong VT, Hayashi M, Tadono T, Nasahara KN (2020). New jaxa high-resolution land use/land cover map for vietnam aiming for natural forest and plantation forest monitoring. Remote Sensing.

[42] Phan DC (2021). First comprehensive quantification of annual land use/cover from 1990 to 2020 across mainland vietnam. Sci. Rep..

[43] Fan P (2019). Urbanization, economic development, environmental and social changes in transitional economies: Vietnam after doimoi. Landsc. Urban Plan..

[44] Meyfroidt P, Lambin EF (2008). The causes of the reforestation in vietnam. Land Use Policy.

[45] McElwee, P. Reforesting “bare hills” in vietnam: Social and environmental consequences of the 5 million hectare reforestation program. *Ambio: A J. Human Environ.* **38**, 325–333 (2009). 10.1579/08-r-520.1 19860156

[46] European Union/ESA/Copernicus. Sentinel-2 msi: Multispectral instrument, level-2a. https://developers.google.com/earth-engine/datasets/catalog/COPERNICUS_S2_SR [Accessed: August 1, 2023].

[47] Gorelick N (2017). Google earth engine: Planetary-scale geospatial analysis for everyone. Remote Sens. Environ..

[48] Japan Aerospace Agency, Earth Observation Research Center (JAXA-EORC). “high-resolution land use and land cover map of mainland vietnam” (2020). https://www.eorc.jaxa.jp/ALOS/en/dataset/lulc/lulc_vnm_v2006_e.htm [Accessed: August 29, 2023].

[49] Truong VT, Phan CD, Nasahara KN, Tadono T (2019). How does land use/land cover map’s accuracy depend on number of classification classes?. SOLA.

[50] Venter ZS, Barton DN, Chakraborty T, Simensen T, Singh G (2022). Global 10 m land use land cover datasets: A comparison of dynamic world, world cover and esri land cover. Remote Sensing.

[51] Sun Z, Xu R, Du W, Wang L, Lu D (2019). High-resolution urban land mapping in china from sentinel 1a/2 imagery based on google earth engine. Remote Sensing.

[52] Salem A, Hashemi-Beni L (2022). Inundated vegetation mapping using sar data: A comparison of polarization configurations of uavsar l-band and sentinel c-band. Remote Sensing.

[53] Kraatz S (2021). Comparison between dense l-band and c-band synthetic aperture radar (sar) time series for crop area mapping over a nisar calibration-validation site. Agronomy.

[54] Zhou Y (2022). For-backward lstm-based missing data reconstruction for time-series landsat images. GIScience & Remote Sensing.

[55] Tahsin S, Medeiros SC, Hooshyar M, Singh A (2017). Optical cloud pixel recovery via machine learning. Remote Sensing.

[56] The Socialist Republic of Vietnam. The national action plan for the implementation of the 2030 sustainable development agenda (issued in conjunction with decision no. 622/qd-ttg dated 10th may 2017 by the prime minister) (2017). https://vietnam.un.org/en/4123-national-action-plan-implementation-2030-sustainable-development-agenda [Accessed: September 3, 2023].

[57] Nguyen, L. & Sarah, G. Vietnam: National strategy on climate change and the action plan on methane emissions reduction. Tech. Rep., United States Department of Agriculture, Foreign Agricultural Service (2022). https://www.fas.usda.gov/data/vietnam-vietnam-issues-national-strategy-climate-change-2050-and-action-plan-methane-emissions [Accessed: September 3, 2023].

[58] The Socialist Republic of Vietnam. Decision on approval of the national action programme on the reduction of green-house gas emissions through the reduction of deforestation and forest degradation, sustainable management of forest resources, and conservation and enhancement of forest carbon stocks (redd+) by 2030 (2017). https://faolex.fao.org/docs/pdf/vie189912.pdf [Accessed: September 3, 2023].

[59] Cochran, W. G. *Sampling techniques* (John Wiley & Sons, 1977).

[60] Olofsson P (2014). Good practices for estimating area and assessing accuracy of land change. Remote Sens. Environ..

[61] Main-Knorn, M. *et al.* Sen2cor for sentinel-2. In *Image and Signal Processing for Remote Sensing XXIII*, vol. 10427, 37–48 (SPIE, 2017).

[62] European Union/ESA/Copernicus. Sentinel-1 sar grd: C-band synthetic aperture radar ground range detected, log scaling. https://developers.google.com/earth-engine/datasets/catalog/COPERNICUS_S1_GRD [Accessed: July 24, 2023].

[63] Vollrath A, Mullissa A, Reiche J (2020). Angular-based radiometric slope correction for sentinel-1 on google earth engine. Remote Sensing.

[64] Hird JN, DeLancey ER, McDermid GJ, Kariyeva J (2017). Google earth engine, open-access satellite data, and machine learning in support of large-area probabilistic wetland mapping. Remote Sensing.

[65] Ottinger M, Kuenzer C (2020). Spaceborne l-band synthetic aperture radar data for geoscientific analyses in coastal land applications: A review. Remote Sensing.

[66] Japan Aerospace Agency, Earth Observation Research Center (JAXA-EORC). Palsar-2 scansar level 2.2. https://developers.google.com/earth-engine/datasets/catalog/JAXA_ALOS_PALSAR-2_Level2_2_ScanSAR [Accessed: August 2, 2023].

[67] Shimada M, Isoguchi O, Tadono T, Isono K (2009). Palsar radiometric and geometric calibration. IEEE Trans. Geosci. Remote Sens..

[68] Hurskainen P, Adhikari H, Siljander M, Pellikka P, Hemp A (2019). Auxiliary datasets improve accuracy of object-based land use/land cover classification in heterogeneous savanna landscapes. Remote Sens. Environ..

[69] Lee DG, Shin YH, Lee D-C (2020). Land cover classification using segnet with slope, aspect, and multidirectional shaded relief images derived from digital surface model. J. Sensors.

[70] Al-Najjar, H., Kalantar, B., Pradhan, B. *et al.* Land cover classification from fused dsm and uav images using convolutional neural networks. remote sens 11: 1–18 (2019).

[71] Schultz M, Voss J, Auer M, Carter S, Zipf A (2017). Open land cover from openstreetmap and remote sensing. Int. J. Appl. Earth Obs. Geoinf..

[72] Japan Aerospace Agency, Earth Observation Research Center (JAXA EORC). Alos global digital surface model “alos world 3d - 30m (aw3d30)”. https://www.eorc.jaxa.jp/ALOS/en/dataset/aw3d30/aw3d30_e.htm [Accessed: August 2, 2023].

[73] Geofabrik. Aopenstreetmap data extracts. http://download.geofabrik.de/ [Accessed: August 2, 2023].

