# Supplementary material for: JAXA’s new high-resolution land use land cover map for Vietnam using a time-feature convolutional neural network
Source: Sci Rep. 2024 Feb 16;14:3926. doi: 10.1038/s41598-024-54308-1 (PMC10873389; doi:10.1038/s41598-024-54308-1)
Supplement: Supplementary file 1 — Supplementary Information. [file 41598_2024_54308_MOESM1_ESM.pdf]

JAXA's New High-Resolution Land Use Land Cover Map for Vietnam Using a Time-Feature Convolutional Neural Network

Van Thinh Truong[1*], Sota Hirayama[2], Duong Cao Phan[3,4], Thanh Tung Hoang[5], Takeo Tadono[2], and Kenlo Nishida Nasahara[6.]

[1]Degree Programs in Life and Earth Sciences, Graduate School of Science and Technology, University of Tsukuba, Tennoudai 1-1-1, Tsukuba, Ibaraki 305-8572, Japan
[2]Earth Observation Research Center (EORC), Japan Aerospace Exploration Agency (JAXA), Sengen 2-1-1, Tsukuba, Ibaraki 305-8505, Japan
[3]Ireland's Centre For Applied AI, School of Computer Science, University College Dublin, Belfield, Dublin 4, D02 V2N9, Ireland
[4]Hydraulic Construction Institute, Vietnam Academy for Water Resources, No. 3, Alley 95, Chua Boc Street, Dong Da district, Hanoi 116765, Vietnam
[5]Faculty of International Studies, Hanoi University, Km 9, Nguyen Trai Road, Nam Tu Liem District, Hanoi 100000, Vietnam
[6]Faculty of Life and Environmental Sciences, University of Tsukuba, Tennoudai 1-1-1, Tsukuba, Ibaraki 305-8572, Japan

*s2036038@s.tsukuba.ac.jp

Supplementary Table S1: Error matrix of sample count for LULC map in the case 1 (optical image only)

		Validation												
		1	2	3	4	5	6	7	8	9	10	11	12	TOTAL
Classified	1	23	0	1	0	0	0	0	0	0	0	1	1	26
	2	0	24	0	1	4	4	1	2	0	0	0	1	37
	3	1	2	67	3	1	2	1	0	0	0	0	0	77
	4	0	2	1	19	2	1	0	0	0	1	0	0	26
	5	0	0	1	0	27	2	0	1	1	2	0	0	34
	6	0	2	1	1	2	64	0	2	1	6	0	0	79
	7	0	0	1	0	2	0	15	0	0	1	0	0	19
	8	0	0	0	0	5	1	1	145	2	11	0	0	165
	9	0	0	0	0	1	1	0	0	15	1	0	0	18
	10	0	0	0	0	4	3	0	11	1	77	0	0	96
	11	0	0	0	1	0	0	0	0	0	1	8	1	11
	12	0	0	0	0	0	0	0	0	0	0	1	11	12
	TOTAL	24	30	72	25	48	78	18	161	20	100	10	14	600

Supplementary Table S2: Error matrix of estimated area proportion for LULC map in the case 1 (optical image only)

		Validation													
		1	2	3	4	5	6	7	8	9	10	11	12	TOTAL	User's accuracy (%)
Classified	1	0.0300	0.0000	0.0013	0.0000	0.0000	0.0000	0.0000	0.0000	0.0000	0.0000	0.0013	0.0013	0.0339	88.5 ± 6.4
	2	0.0000	0.0346	0.0000	0.0014	0.0058	0.0058	0.0014	0.0029	0.0000	0.0000	0.0000	0.0014	0.0534	64.9 ± 8.0
	3	0.0016	0.0033	0.1096	0.0049	0.0016	0.0033	0.0016	0.0000	0.0000	0.0000	0.0000	0.0000	0.1260	87.0 ± 3.9
	4	0.0000	0.0032	0.0016	0.0305	0.0032	0.0016	0.0000	0.0000	0.0000	0.0016	0.0000	0.0000	0.0417	73.1 ± 8.9
	5	0.0000	0.0000	0.0017	0.0000	0.0447	0.0033	0.0000	0.0017	0.0017	0.0033	0.0000	0.0000	0.0563	79.4 ± 7.0
	6	0.0000	0.0033	0.0017	0.0017	0.0033	0.1060	0.0000	0.0033	0.0017	0.0099	0.0000	0.0000	0.1309	81.0 ± 4.4
	7	0.0000	0.0000	0.0014	0.0000	0.0029	0.0000	0.0217	0.0000	0.0000	0.0014	0.0000	0.0000	0.0275	78.9 ± 9.6
	8	0.0000	0.0000	0.0000	0.0000	0.0087	0.0017	0.0017	0.2530	0.0035	0.0192	0.0000	0.0000	0.2879	87.9 ± 2.5
	9	0.0000	0.0000	0.0000	0.0000	0.0016	0.0016	0.0000	0.0000	0.0247	0.0016	0.0000	0.0000	0.0296	83.3 ± 9.0
	10	0.0000	0.0000	0.0000	0.0000	0.0072	0.0054	0.0000	0.0198	0.0018	0.1385	0.0000	0.0000	0.1727	80.2 ± 4.1
	11	0.0000	0.0000	0.0000	0.0013	0.0000	0.0000	0.0000	0.0000	0.0000	0.0013	0.0106	0.0013	0.0145	72.7 ± 14.1
	12	0.0000	0.0000	0.0000	0.0000	0.0000	0.0000	0.0000	0.0000	0.0000	0.0000	0.0021	0.0235	0.0256	91.7 ± 8.3
	TOTAL	0.0317	0.0444	0.1173	0.0398	0.0791	0.1288	0.0265	0.2806	0.0333	0.1770	0.0140	0.0276	1	---
	Producer's accuracy (%)	94.8 ± 4.9	78.0 ± 7.3	93.5 ± 2.8	76.6 ± 7.6	56.5 ± 5.7	82.3 ± 3.9	81.8 ± 8.8	90.1 ± 2.1	74.1 ± 8.8	78.3 ± 3.6	75.4 ± 13.9	85.2 ± 7.4	---	Overall acc. 82.7 ± 1.5

Supplementary Table S3: Error matrix of sample count for LULC map in the case 2 (SAR image only)

		Validation												
		1	2	3	4	5	6	7	8	9	10	11	12	TOTAL
Classified	1	24	0	1	1	0	0	1	0	0	0	0	1	28
	2	0	22	0	0	0	3	1	1	0	1	0	0	28
	3	0	0	63	2	0	1	0	0	0	0	0	1	67
	4	0	2	2	17	2	3	0	1	1	1	0	0	29
	5	0	0	0	0	19	0	2	1	1	1	0	0	24
	6	0	3	3	3	3	57	0	3	2	5	0	0	79
	7	0	0	2	0	4	0	13	1	0	1	0	1	22
	8	0	0	0	0	15	1	1	144	3	6	0	0	170
	9	0	0	0	0	1	3	0	0	12	0	0	0	16
	10	0	1	0	2	2	10	0	10	1	85	0	0	111
	11	0	2	0	0	2	0	0	0	0	0	7	0	11
	12	0	0	1	0	0	0	0	0	0	0	3	11	15
	TOTAL	24	30	72	25	48	78	18	161	20	100	10	14	600

Supplementary Table S4: Error matrix of estimated area proportion for LULC map in the case 2 (SAR image only)

		Validation													
		1	2	3	4	5	6	7	8	9	10	11	12	TOTAL	User's accuracy (%)
Classified	1	0.0291	0.0000	0.0012	0.0012	0.0000	0.0000	0.0012	0.0000	0.0000	0.0000	0.0000	0.0012	0.0339	85.7 ± 6.7
	2	0.0000	0.0420	0.0000	0.0000	0.0000	0.0057	0.0019	0.0019	0.0000	0.0019	0.0000	0.0000	0.0534	78.6 ± 7.9
	3	0.0000	0.0000	0.1185	0.0038	0.0000	0.0019	0.0000	0.0000	0.0000	0.0000	0.0000	0.0019	0.1260	94.0 ± 2.9
	4	0.0000	0.0029	0.0029	0.0245	0.0029	0.0043	0.0000	0.0014	0.0014	0.0014	0.0000	0.0000	0.0417	58.6 ± 9.3
	5	0.0000	0.0000	0.0000	0.0000	0.0445	0.0000	0.0047	0.0023	0.0023	0.0023	0.0000	0.0000	0.0563	79.2 ± 8.5
	6	0.0000	0.0050	0.0050	0.0050	0.0050	0.0944	0.0000	0.0050	0.0033	0.0083	0.0000	0.0000	0.1309	72.2 ± 5.1
	7	0.0000	0.0000	0.0025	0.0000	0.0050	0.0000	0.0162	0.0012	0.0000	0.0012	0.0000	0.0012	0.0275	59.1 ± 10.7
	8	0.0000	0.0000	0.0000	0.0000	0.0254	0.0017	0.0017	0.2438	0.0051	0.0102	0.0000	0.0000	0.2879	84.7 ± 2.8
	9	0.0000	0.0000	0.0000	0.0000	0.0018	0.0055	0.0000	0.0000	0.0222	0.0000	0.0000	0.0000	0.0296	75.0 ± 11.2
	10	0.0000	0.0016	0.0000	0.0031	0.0031	0.0156	0.0000	0.0156	0.0016	0.1322	0.0000	0.0000	0.1727	76.6 ± 4.0
	11	0.0000	0.0026	0.0000	0.0000	0.0026	0.0000	0.0000	0.0000	0.0000	0.0000	0.0092	0.0000	0.0145	63.6 ± 15.2
	12	0.0000	0.0000	0.0017	0.0000	0.0000	0.0000	0.0000	0.0000	0.0000	0.0000	0.0051	0.0188	0.0256	73.3 ± 11.8
	TOTAL	0.0291	0.0540	0.1317	0.0375	0.0904	0.1292	0.0257	0.2713	0.0359	0.1576	0.0144	0.0231	1	---
	Producer's accuracy (%)	100	77.7 ± 6.3	89.9 ± 3.0	65.2 ± 8.8	49.3 ± 5.2	73.1 ± 4.4	63.1 ± 11.4	89.9 ± 2.2	61.8 ± 9.1	83.9 ± 3.5	64.3 ± 13.4	81.2 ± 9.3	---	Overall acc. 79.6 ± 1.6

Supplementary Table S5: Error matrix of sample count for LULC map in the case 3 (optical image + SAR image)

		Validation												
		1	2	3	4	5	6	7	8	9	10	11	12	TOTAL
Classified	1	23	0	0	1	0	0	0	0	0	0	0	1	25
	2	0	28	0	0	0	1	0	1	0	1	0	0	31
	3	1	1	68	1	0	1	0	0	0	1	0	0	73
	4	0	1	1	22	1	1	0	0	0	0	0	0	26
	5	0	0	1	0	41	0	0	4	1	1	0	0	48
	6	0	0	1	1	2	70	0	0	0	3	0	0	77
	7	0	0	1	0	0	0	17	0	0	1	0	0	19
	8	0	0	0	0	3	3	0	147	1	5	0	0	159
	9	0	0	0	0	0	0	0	0	17	1	0	0	18
	10	0	0	0	0	0	2	1	9	1	87	0	0	100
	11	0	0	0	0	1	0	0	0	0	0	9	0	10
	12	0	0	0	0	0	0	0	0	0	0	1	13	14
	TOTAL	24	30	72	25	48	78	18	161	20	100	10	14	600

Supplementary Table S6: Error matrix of estimated area proportion for LULC map in the case 3 (optical image + SAR image)

		Validation													
		1	2	3	4	5	6	7	8	9	10	11	12	TOTAL	User's accuracy (%)
Classified	1	0.0312	0.0000	0.0000	0.0014	0.0000	0.0000	0.0000	0.0000	0.0000	0.0000	0.0000	0.0014	0.0339	92.0 ± 5.5
	2	0.0000	0.0482	0.0000	0.0000	0.0000	0.0017	0.0000	0.0017	0.0000	0.0017	0.0000	0.0000	0.0534	90.3 ± 5.4
	3	0.0017	0.0017	0.1173	0.0017	0.0000	0.0017	0.0000	0.0000	0.0000	0.0017	0.0000	0.0000	0.1260	93.2 ± 3.0
	4	0.0000	0.0016	0.0016	0.0353	0.0016	0.0016	0.0000	0.0000	0.0000	0.0000	0.0000	0.0000	0.0417	84.6 ± 7.2
	5	0.0000	0.0000	0.0012	0.0000	0.0481	0.0000	0.0000	0.0047	0.0012	0.0012	0.0000	0.0000	0.0563	85.4 ± 5.1
	6	0.0000	0.0000	0.0017	0.0017	0.0034	0.1190	0.0000	0.0000	0.0000	0.0051	0.0000	0.0000	0.1309	90.9 ± 3.3
	7	0.0000	0.0000	0.0014	0.0000	0.0000	0.0000	0.0246	0.0000	0.0000	0.0014	0.0000	0.0000	0.0275	89.5 ± 7.2
	8	0.0000	0.0000	0.0000	0.0000	0.0054	0.0054	0.0000	0.2661	0.0018	0.0091	0.0000	0.0000	0.2879	92.5 ± 2.1
	9	0.0000	0.0000	0.0000	0.0000	0.0000	0.0000	0.0000	0.0000	0.0279	0.0016	0.0000	0.0000	0.0296	94.4 ± 5.6
	10	0.0000	0.0000	0.0000	0.0000	0.0000	0.0035	0.0017	0.0155	0.0017	0.1502	0.0000	0.0000	0.1727	87.0 ± 3.4
	11	0.0000	0.0000	0.0000	0.0000	0.0015	0.0000	0.0000	0.0000	0.0000	0.0000	0.0131	0.0000	0.0145	90.0 ± 10.0
	12	0.0000	0.0000	0.0000	0.0000	0.0000	0.0000	0.0000	0.0000	0.0000	0.0000	0.0018	0.0238	0.0256	92.9 ± 7.1
	TOTAL	0.0330	0.0516	0.1233	0.0401	0.0599	0.1329	0.0263	0.2881	0.0327	0.1721	0.0149	0.0252	1	---
	Producer's accuracy (%)	94.7 ± 5.0	93.5 ± 4.3	95.2 ± 2.3	88.1 ± 6.2	80.1 ± 6.1	89.5 ± 3.3	93.4 ± 6.2	92.3 ± 1.8	85.6 ± 7.3	87.3 ± 3.1	87.7 ± 10.8	94.6 ± 5.1	---	Overall acc. 90.5 ± 1.2

Supplementary Table S7: Error matrix of sample count for the reclassified LULC map in the case 3 (optical image + SAR image) with 7 categories

		Validation							
		1	2	3	4	5	6	7	TOTAL
Classified	1	37	0	0	0	0	1	1	39
	2	0	28	3	0	0	0	0	31
	3	0	0	346	1	5	0	2	354
	4	0	0	1	17	0	0	1	19
	5	0	0	6	0	41	0	1	48
	6	0	0	0	0	1	9	0	10
	7	1	2	3	0	1	0	92	99
	TOTAL	38	30	359	18	48	10	97	600

Supplementary Table S8: Error matrix of estimated area proportion for reclassified LULC map in the case 3 (optical image + SAR image), with 7 categories

		Validation								
		1	2	3	4	5	6	7	TOTAL	User's accuracy (%)
Classified	1	0.0565	0.0000	0.0000	0.0000	0.0000	0.0015	0.0015	0.0596	94.9 ± 3.6
	2	0.0000	0.0482	0.0052	0.0000	0.0000	0.0000	0.0000	0.0534	90.3 ± 5.4
	3	0.0000	0.0000	0.1639	0.0005	0.0024	0.0000	0.0009	0.1677	97.7 ± 0.8
	4	0.0000	0.0000	0.0014	0.0246	0.0000	0.0000	0.0014	0.0275	89.5 ± 7.2
	5	0.0000	0.0000	0.0070	0.0000	0.0481	0.0000	0.0012	0.0563	85.4 ± 5.1
	6	0.0000	0.0000	0.0000	0.0000	0.0015	0.0131	0.0000	0.0145	90.0 ± 10.0
	7	0.0063	0.0125	0.0188	0.0000	0.0063	0.0000	0.5771	0.6210	92.9 ± 2.6
	TOTAL	0.0628	0.0608	0.1964	0.0251	0.0582	0.0146	0.5822	1	---
	Producer's accuracy (%)	90.0 ± 9.0	79.4 ± 11.6	83.5 ± 4.9	98.1 ± 1.9	82.6 ± 9.3	89.5 ± 9.4	99.1 ± 0.4	---	Overall acc. 93.2 ± 1.7

Supplementary Table S9: Error matrix of sample count for the reclassified LULC map of Dynamic World with 7 categories

		Validation							
		1	2	3	4	5	6	7	TOTAL
Classified	1	28	0	0	0	0	1	2	31
	2	1	29	12	2	1	1	8	54
	3	0	0	315	2	33	5	9	364
	4	0	0	1	9	0	0	2	12
	5	0	0	23	4	13	0	4	44
	6	8	0	0	0	0	3	0	11
	7	1	1	8	1	1	0	72	84
	TOTAL	38	30	359	18	48	10	97	600

Supplementary Table S10: Error matrix of estimated area proportion for reclassified LULC map of Dynamic World with 7 categories

		Validation								
		1	2	3	4	5	6	7	TOTAL	User's accuracy (%)
Classified	1	0.0565	0.0000	0.0000	0.0000	0.0000	0.0015	0.0015	0.0596	94.9 ± 3.6
	2	0.0000	0.0482	0.0052	0.0000	0.0000	0.0000	0.0000	0.0534	90.3 ± 5.4
	3	0.0000	0.0000	0.1639	0.0005	0.0024	0.0000	0.0009	0.1677	97.7 ± 0.8
	4	0.0000	0.0000	0.0014	0.0246	0.0000	0.0000	0.0014	0.0275	89.5 ± 7.2
	5	0.0000	0.0000	0.0070	0.0000	0.0481	0.0000	0.0012	0.0563	85.4 ± 5.1
	6	0.0000	0.0000	0.0000	0.0000	0.0015	0.0131	0.0000	0.0145	90.0 ± 10.0
	7	0.0063	0.0125	0.0188	0.0000	0.0063	0.0000	0.5771	0.6210	92.9 ± 2.6
	TOTAL	0.0628	0.0608	0.1964	0.0251	0.0582	0.0146	0.5822	1	---
	Producer's accuracy (%)	90.0 ± 9.0	79.4 ± 11.6	83.5 ± 4.9	98.1 ± 1.9	82.6 ± 9.3	89.5 ± 9.4	99.1 ± 0.4	---	Overall acc. 93.2 ± 1.7

Supplementary Table S11: Error matrix of sample count for the reclassified LULC map of ESRI with 7 categories

		Validation							
		1	2	3	4	5	6	7	TOTAL
Classified	1	34	0	0	0	0	3	0	37
	2	0	30	17	3	2	1	11	64
	3	0	0	278	1	26	2	4	311
	4	0	0	0	6	0	0	0	6
	5	2	0	34	8	19	0	7	70
	6	2	0	0	0	0	3	0	5
	7	0	0	30	0	1	1	75	107
	TOTAL	38	30	359	18	48	10	97	600

Supplementary Table S12: Error matrix of estimated area proportion for reclassified LULC map of ESRI with 7 categories

		Validation								
		1	2	3	4	5	6	7	TOTAL	User's accuracy (%)
Classified	1	0.0547	0.0000	0.0000	0.0000	0.0000	0.0048	0.0000	0.0596	91.9 ± 4.5
	2	0.0000	0.0250	0.0142	0.0025	0.0017	0.0008	0.0092	0.0534	46.9 ± 6.3
	3	0.0000	0.0000	0.1499	0.0005	0.0140	0.0011	0.0022	0.1677	89.4 ± 1.7
	4	0.0000	0.0000	0.0000	0.0275	0.0000	0.0000	0.0000	0.0275	100.0 ± 0.0
	5	0.0016	0.0000	0.0273	0.0064	0.0153	0.0000	0.0056	0.0563	27.1 ± 5.4
	6	0.0058	0.0000	0.0000	0.0000	0.0000	0.0087	0.0000	0.0145	60.0 ± 24.5
	7	0.0000	0.0000	0.1741	0.0000	0.0058	0.0058	0.4353	0.6210	70.1 ± 4.4
	TOTAL	0.0622	0.0250	0.3656	0.0369	0.0368	0.0213	0.4523	1	---
	Producer's accuracy (%)	88.1 ± 5.3	100.0 ± 0.0	41.0 ± 3.1	74.4 ± 5.3	41.5 ± 8.8	41.0 ± 16.0	96.2 ± 0.8	---	Overall acc. 71.6 ± 2.9

Supplementary Table S13: Error matrix of sample count for the reclassified LULC map of ESA with 7 categories

		Validation							
		1	2	3	4	5	6	7	TOTAL
Classified	1	26	0	0	0	0	0	0	26
	2	0	15	0	1	0	0	1	17
	3	1	7	324	2	28	1	16	379
	4	3	5	0	9	0	1	2	20
	5	4	2	31	6	19	0	3	65
	6	2	0	0	0	0	7	1	10
	7	2	1	4	0	1	1	74	83
	TOTAL	38	30	359	18	48	10	97	600

Supplementary Table S14: Error matrix of estimated area proportion for reclassified LULC map of ESA with 7 categories.

		Validation								
		1	2	3	4	5	6	7	TOTAL	User's accuracy (%)
Classified	1	0.0596	0.0000	0.0000	0.0000	0.0000	0.0000	0.0000	0.0596	100.0 ± 0.0
	2	0.0000	0.0471	0.0000	0.0031	0.0000	0.0000	0.0031	0.0534	88.2 ± 8.1
	3	0.0004	0.0031	0.1434	0.0009	0.0124	0.0004	0.0071	0.1677	85.5 ± 1.8
	4	0.0041	0.0069	0.0000	0.0124	0.0000	0.0014	0.0027	0.0275	45 ± 11.4
	5	0.0035	0.0017	0.0268	0.0052	0.0164	0.0000	0.0026	0.0563	29.2 ± 5.7
	6	0.0029	0.0000	0.0000	0.0000	0.0000	0.0102	0.0015	0.0145	70.0 ± 15.3
	7	0.0150	0.0075	0.0299	0.0000	0.0075	0.0075	0.5537	0.6210	89.2 ± 3.4
	TOTAL	0.0855	0.0663	0.2001	0.0216	0.0363	0.0195	0.5707	1	---
	Producer's accuracy (%)	69.7 ± 9.0	71.1 ± 8.9	71.6 ± 5.4	57.3 ± 11.8	45.3 ± 10.9	52.2 ± 21.2	97.0 ± 0.8	---	Overall acc. 84.3 ± 2.3

Supplementary Table S15: Error matrix of sample count for the reclassified LULC map of JAXA in 2016 with 7 categories

		Validation							
		1	2	3	4	5	6	7	TOTAL
Classified	1	141	2	0	3	0	0	4	150
	2	0	134	6	4	3	0	3	150
	3	4	9	1088	26	26	0	47	1200
	4	0	6	2	111	18	0	13	150
	5	0	1	45	2	236	0	16	300
	6	3	0	2	0	0	139	6	150
	7	25	18	4	0	4	0	549	600
	TOTAL	173	170	1147	146	287	139	638	2700

Supplementary Table S16: Error matrix of estimated area proportion for reclassified LULC map of JAXA in 2016 with 7 categories.

		Validation								
		1	2	3	4	5	6	7	TOTAL	User's accuracy (%)
Classified	1	0.0306	0.0004	0.0000	0.0007	0.0000	0.0000	0.0009	0.0326	94.0 ± 1.9
	2	0.0000	0.0223	0.0010	0.0007	0.0005	0.0000	0.0005	0.0250	89.3 ± 2.5
	3	0.0017	0.0038	0.4558	0.0109	0.0109	0.0000	0.0197	0.5028	90.7 ± 0.8
	4	0.0000	0.0017	0.0006	0.0320	0.0052	0.0000	0.0038	0.0433	74 ± 3.6
	5	0.0000	0.0005	0.0225	0.0010	0.1179	0.0000	0.0080	0.1499	78.7 ± 2.4
	6	0.0001	0.0000	0.0001	0.0000	0.0000	0.0069	0.0003	0.0074	92.7 ± 2.1
	7	0.0099	0.0072	0.0016	0.0000	0.0016	0.0000	0.2182	0.2384	91.5 ± 1.1
	TOTAL	0.0424	0.0359	0.4816	0.0452	0.1361	0.0069	0.2513	1	---
	Producer's accuracy (%)	72.3 ± 3.6	62.1 ± 4.0	94.7 ± 0.6	70.8 ± 3.7	86.6 ± 1.7	100 ± 0.0	86.8 ± 1.3	---	Overall acc. 88.4 ± 0.6
